# Unraveling RubisCO Form I and Form II Regulation in an Uncultured Organism from a Deep-Sea Hydrothermal Vent via Metagenomic and Mutagenesis Studies

**DOI:** 10.3389/fmicb.2017.01303

**Published:** 2017-07-12

**Authors:** Stefanie Böhnke, Mirjam Perner

**Affiliations:** Molecular Biology of Microbial Consortia, Biocenter Klein Flottbek, University of Hamburg Hamburg, Germany

**Keywords:** autotrophic CO_2_ fixation, Calvin-Benson-Bassham (CBB) cycle, RubisCO gene regulation, LysR, CbbQ, CbbO, heterologous gene expression, non-native system

## Abstract

Ribulose-1,5-bisphosphate carboxylase/oxygenase (RubisCO) catalyzes the first major step of carbon fixation in the Calvin-Benson-Bassham (CBB) cycle. This autotrophic CO_2_ fixation cycle accounts for almost all the assimilated carbon on Earth. Due to the primary role that RubisCO plays in autotrophic carbon fixation, it is important to understand how its gene expression is regulated and the enzyme is activated. Since the majority of all microorganisms are currently not culturable, we used a metagenomic approach to identify genes and enzymes associated with RubisCO expression. The investigated metagenomic DNA fragment originates from the deep-sea hydrothermal vent field Nibelungen at 8°18′ S along the Mid-Atlantic Ridge. It is 13,046 bp and resembles genes from *Thiomicrospira crunogena*. The fragment encodes nine open reading frames (ORFs) which include two types of RubisCO, form I (CbbL/S) and form II (CbbM), two LysR transcriptional regulators (LysR1 and LysR2), two von Willebrand factor type A (CbbO-m and CbbO-1), and two AAA+ ATPases (CbbQ-m and CbbQ-1), expected to function as RubisCO activating enzymes. *In silico* analyses uncovered several putative LysR binding sites and promoter structures. Functions of some of these DNA motifs were experimentally confirmed. For example, according to mobility shift assays LysR1’s binding ability to the intergenic region of *lysR1* and *cbbL* appears to be intensified when CbbL or LysR2 are present. Binding of LysR2 upstream of *cbbM* appears to be intensified if CbbM is present. Our study suggests that CbbQ-m and CbbO-m activate CbbL and that LysR1 and LysR2 proteins promote CbbQ-m/CbbO-m expression. CbbO-1 seems to activate CbbM and CbbM itself appears to contribute to intensifying LysR’s binding ability and thus its own transcriptional regulation. CbbM furthermore appears to impair *cbbL* expression. A model summarizes the findings and predicts putative interactions of the different proteins influencing RubisCO gene regulation and expression.

## Introduction

Ribulose-1,5-bisphosphate carboxylase/oxygenase (RubisCO, EC 4.1.1.39) is believed to be the most abundant enzyme on Earth (Ellis, 1979; Raven, 2009). It is the key enzyme of the autotrophic Calvin-Benson-Bassham (CBB) cycle and catalyzes the carboxylation of ribulose-1,5-bisphosphate (RuBP) to 3-phosphoglycerate (3-PGA) (Berg, 2011). Since the CBB cycle is estimated to account for most of Earth’s net primary production (>99.5% of 105 × 10^9^ tons/year) (Field et al., 1998; Raven, 2013), it is important to understand RubisCO expression and its activation in many different organisms.

The RubisCO enzyme is widespread and can be found in plants, algae, cyanobacteria, many autotrophic bacteria (phototrophs and chemolithotrophs), and archaea (Tabita et al., 2007; Hauser et al., 2015). Although four types of structural RubisCOs are known, only the RubisCO form I (CbbLS) and the form II (CbbM) are evidenced to operate in the classical CBB cycle (Berg, 2011). For the expression and activation of a catalytically active form I and form II RubisCO distinct transcriptional regulators and activases are essential (Maddocks and Oyston, 2008; Dangel and Tabita, 2015; Tsai et al., 2015). LysR-type transcriptional regulators (LTTRs) have been found adjacent to the structural RubisCO genes in several genomes and are evidenced to regulate their transcription (Dangel and Tabita, 2015). LTTRs can function as an activator and/or as a repressor for their target genes (Maddocks and Oyston, 2008 and references therein), but can also positively autoregulate their own transcription (Axler-DiPerte et al., 2006). Indeed, LTTR associated regulation can be highly complex as is indicated by LTTRs which need to interact with other transcriptional regulators (Joshi et al., 2013; Dangel et al., 2014). Since RubisCO forms inhibited complexes with its substrate RuBP but also with other sugar phosphates (Tsai et al., 2015), the removal of the active site inhibitor is essential for proceeding with the RubisCO catalyzed carboxylation reaction. In case of plant green-type and α-proteobacterial red-type form I RubisCOs, this is done by the RubisCO activase (rca) and CbbX, respectively (Parry et al., 2008; Mueller-Cajar et al., 2011). CbbQ (AAA+ATPase) and CbbO (von Willebrand factor type A) represent a third class of RubisCO activases and were shown to act on green-type form I RubisCOs of chemoautotrophic bacteria (Tsai et al., 2015).

Given that the majority of microorganisms are currently unculturable (Amann et al., 1995), we recently developed an activity-based screen, which enables us to seek RubisCO active clones from metagenomic fosmid libraries (Böhnke and Perner, 2015). One of these newly discovered RubisCO active metagenomic clones stems from a fosmid library constructed with DNA from the Nibelungen vent field (8°18′S on the Mid-Atlantic Ridge): It exhibited similarities to genes from the gammaproteobacterial *Thiomicrospira crunogena* XCL-2 (96%). Our metagenomic fragment encodes a 13 kb RubisCO gene cluster and flanking DNA regions of additional 22.2 kb. The 13 kb DNA fragment encodes two divergently directed reading frames: (i) *lysR1*, *lysR2*, *cbbM*, *cbbQ-m*, and *cbbO-m*, and (ii) *cbbL*, *cbbS*, *cbbQ-1*, and *cbbO*-*1*. To date, only one study has ever investigated regulatory mechanisms in metagenome derived RubisCO gene clusters (Böhnke and Perner, 2015). Here, total RubisCO activity was significantly influenced when *cbbL* and *cbbM* neighboring genes were knocked out (Böhnke and Perner, 2015), but it remained unclear which of the two RubisCOs was primarily affected by these mutations. While most of the studies on RubisCO regulation investigate the regulation of alphaproteobacterial RubisCOs (Paoli et al., 1998; Dubbs and Tabita, 2003; van Keulen et al., 2003; Dubbs et al., 2004; Joshi et al., 2013; Dangel et al., 2014), little work exists on the regulatory machinery behind gammaproteobacterial RubisCO transcription (Kusano and Sugawara, 1993). The arrangement of alphaproteobacterial RubisCOs and their associated genes as well as the location of the RubisCO gene clusters on the genome are very different to what is observed on our metagenomic fragment. For example, while the alphaproteobacterial *Rhodobacter capsulatus* RubisCO form I gene cluster is arranged like our RubisCO form I gene cluster (*lysR1 cbbLSQO*), the RubisCO form II gene cluster is considerably different to that on our metagenomic fragment (*cbbFPTGAM* versus *cbbMQO*, respectively) (Paoli et al., 1998) suggesting different interactions with respect to regulatory processes. Also, our metagenomic RubisCO form I and form II gene clusters are located within each other’s vicinity on a 13 kb DNA fragment. In contrast, the RubisCO gene clusters of the so far investigated *Alphaproteobacteria* are either encoded on different chromosomes (*Rhodobacter sphaeroides*) or on distant regions of the genome (separated by 2 Mb or 1.4 Mb, *Rhodobacter capsulatus* and *Rhodopseudomonas palustris*, respectively) (Paoli et al., 1998; Dubbs and Tabita, 2004; Joshi et al., 2013). The here investigated metagenome derived form I and form II RubisCOs, thus, represent a unique opportunity to investigate the role that genes and respective products have on the expression and activation of two forms of RubisCOs from an uncultured Gammaproteobacterium colonizing a chemically dynamic environment.

## Materials and Methods

### Bacterial Strains, Vectors, and Constructs, Media, and Growth Conditions

The bacterial strains, vectors, and constructs used in this study are summarized in **Table 1**. *Escherichia coli* cultures were routinely grown on lysogeny broth (LB) medium (Bertani, 1951). For cloning procedures cultures were incubated at 37°C. If cultivated for measuring recombinant RubisCO activities, the growth temperature was lowered to 28°C, while cultures grown as part of over expression experiments were incubated at 17°C or 22°C. If required, the following supplements were added: ampicillin, 100 μg ml^-1^; 5-bromo-4-chloro-3-indolyl-β-D-galactopyranoside (X-gal), 50 μg ml^-1^, chloramphenicol, 12.5 μg ml^-1^; isopropyl β-D-1-thiogalactopyranoside (IPTG), 100 μg ml^-1^ (cloning) or 0.1–1 mM (expression); kanamycin, 50 μg ml^-1^, and tetracycline, 10 μg ml^-1^.

**Table 1:** Strains, vectors, and constructs used in this study.

Strain, plasmid or construct	Genotype or characteristics	Size [bp]	Source
Epi300^TM^-T1^R^	F^-^, *mcr*A, Δ(*mrr*-*hsd*RMS-*mcr*BC) Φ80*dlac*ZΔM15, Δ*lac*X74, *rec*A1 *end*A1, *ara*D139, Δ(*ara, leu*)7697, *gal*U, *gal*K, λ^-^, *rps*L, *nup*G, *trf*A, *ton*A, *dhfr*	/	epicentre^®^ (Madison, WI, United States)
*E. coli* Rosetta-gami 2	Δ(*ara*-*leu*)7697 Δ*lac*X74 Δ*pho*A *Pvu*II *pho*R, *ara*D139, *ahp*C, *gal*E, *gal*K, *rps*L (DE3), F′[lac*^+^* lac*I^q^* pro] *gor*522::Tn10 *trx*B *p*RARE2 (Cam^R^, Str^R^, Tet^R^)	/	Novagen/Merck (Darmstadt, Germany)
pCC1FOS^TM^	Fosmid cloning vector, oriV, ori2, *red*F, *rep*E, *par*A, *par*B, *par*C, *cos, lox*P, *lac*Z, Cam^R^, P T7	8,139	epicentre^®^ (Madison, WI, United States)
pet21a	Expression vector, *lac*I, Amp^R^, P T7, C-terminal His 6-tag coding sequence	5,443	Novagen/Merck (Darmstadt, Germany)
71C2	pCC1FOS metagenomic fosmid vector containing a RubisCO gene cluster (*cbbO-mQ-mM lysR2 lysR1 cbbLSQ-1O-1*) and 22.2 kb flanking DNA	35,195	Böhnke and Perner, 2015
71C2II	pCC1FOS vector containing a metagenome derived RubisCO gene cluster (*cbbO-mQ-mM lysR2 lysR1 cbbLSQ-1O-1*) subcloned from 71C2	13,023	Böhnke and Perner, 2015
22IIΔ*cbbM*	Transposon clone based on 71C2II with an insertion in the *cbbM* structural gene at position 171aa of 459aa	14,244	Böhnke and Perner, 2015
24IIΔ*cbbL*	Transposon clone based on 71C2II with an insertion in the *cbbL* structural gene at position 41aa of 472aa	14,244	Böhnke and Perner, 2015
			
pet21a::*cbbL*	*cbbL* cloned from 71C2	1,421	This study
pet21a::*cbbM*	*cbbL* cloned from 71C2	1,386	This study
			
pet21a::*lysR1*	*lysR1* cloned from 71C2	932	This study
pet21a::*lysR2*	*lysR2* cloned from 71C2	950	This study

*Characteristics of double transposon clones constructed in this study are indicated in Supplementary Table 1.*

### Construction of Double Transposon Mutant Libraries

Two double transposon mutant libraries were constructed from two versions of the 13 kb metagenomic fragment consisting of the RubisCO gene cluster (*cbbO-mQ-mM lysR2 lysR1 cbbLSQ-1O-1*; accession: KJ639815.1) using the EZ-Tn5^TM^ <TET-1> Tnp Transposome^TM^ Kit (epicentre^®^, Madison, WI, United States) according to manufacturer’s instructions, with chemically competent Epi300^TM^ – T1^R^ (epicentre^®^) as the host. One library was constructed with transposon clone 22II, where the *cbbM* structural gene was deleted (Δ*cbbM)*. The second library was constructed using transposon clone 24II, where the *cbbL* structural gene was impaired (Δ*cbbL*). Clones containing fosmids with <TET-1> insertions were selected on LB agar plates using the following antibiotic additions: (i) chloramphenicol (12.5 μg ml^-1^) for selecting the fosmid vector, (ii) kanamycin (50 μg ml^-1^) to verify the presence of the first insertion, i.e., Δ*cbbM* or Δ*cbbL*, and (iii) tetracycline (100 μg ml^-1^) to verify the insertion of the second transposon element. Fosmids of double transposon clones were isolated from autoinduced cultures (for detailed information on autoinduction procedure see the manual for the CopyControl^TM^ Fosmid Library Production Kit, epicentre^®^) using the High-Speed Plasmid Mini Kit (Geneaid, New Taipei City, Taiwan) according to manufacturer’s instruction. Isolated fosmids were sequenced starting from the <TET-1> insertion using the TET-1 FP-1 forward and TET-1 RP-1 reverse primers (see manual of the EZ-Tn5^TM^ <TET-1> Insertion Kit, epicentre^®^) to identify the exact insertion position. Selected clones were tested for their RubisCO activities.

### RubisCO Activity Assay

For RubisCO activity measurements double transposon clones were cultivated at 28°C on 200 ml pre-heated LB medium supplemented with chloramphenicol (12.5 μg ml^-1^), kanamycin (50 μg ml^-1^), tetracycline (10 μg ml^-1^), and autoinduction solution [1x final concentration (epicentre^®^)] in 1 l flasks with shaking (130 rpm) and harvested after 18 h by centrifugation (9,800 × *g*, 10 min, and 4°C). Subsequently crude extracts were prepared. For this purpose, cell pellets were washed twice with buffer A [100 mM Tris-HCl (pH 7.8), 10 mM MgCl_2_, 1 mM EDTA, 25 mM NaHCO_3_ and 1 mM DTT] before resuspension in 2 ml of the same buffer. Cells were disrupted by the French pressure cell press method, followed by centrifugation (19,580 × *g*, 20 min, and 4°C), as described before (Böhnke and Perner, 2015). The generated crude extracts were finally used as template to perform the RubisCO activity assay, where the concentrations of the reactant (RuBP) and the product (3-PGA) of RubisCO reaction were quantified over time using High-Performance Liquid Chromatography (HPLC) (Böhnke and Perner, 2015). At least two biological replicates and three technical replicates were used for the RubisCO activity assay. Mean values of technical replicates were used to calculate the overall mean. Errors of RubisCO activity measurements were calculated with the Gaussian propagation of error. Standard derivations of technical replicates were propagated forward and are thus entered into the equation. Significant differences were calculated using an unpaired *t*-test with equal variance and two-tailed distribution. For each performed HPLC run different controls were tested additionally to the measured samples. The crude extract of the metagenome derived fosmid clone 71C2 containing the RubisCO gene cluster (*cbbO-mQ-mM lysR2 lysR1 cbbLSQ-1O-1*) and 22.2 kb flanking DNA serves as positive control and the crude extract of an *E. coli* fosmid clone without RubisCO genes encoded on its fosmid insert serves as a negative control. A protein free reference sample with 5 mM RuBP and 5 mM 3-PGA dissolved in buffer A were furthermore applied through the assay and used (i) for sample peak assignment and (ii) to gather the non-enzymatic degradation of educts and products. The latter was used to calculate the pseudo-activity which is subtracted from each sample activity.

### Quantitative Reverse Transcriptase PCR

Clones were cultivated in 100 ml flasks on 20 ml LB media supplemented with autoinduction solution [1x final concentration (epicentre^®^)] and the following antibiotics: chloramphenicol (12.5 μg ml^-1^) for the fosmid subclone 71C2II, chloramphenicol (12.5 μg ml^-1^) and kanamycin (50 μg ml^-1^) for transposon clones 22II (Δ*cbbM*), 24II (Δ*cbbL*), 6II (Δ*lysR1*), and 149II (Δ*lysR2*), and chloramphenicol (12.5 μg ml^-1^), kanamycin (50 μg ml^-1^), and tetracycline (10 μg ml^-1^) for the double transposon clones 22II2B2 (Δ*cbbM* Δ*lysR1*), 22II3A3 (Δ*cbbM* Δ*lysR2*), 24II1H1 (Δ*cbbL* Δ*lysR1*), and 24II1H7 (Δ*cbbL* Δ*lysR2*). Cultures were allowed to grow until an optical density (λ = 600 nm) between 2.0 and 3.0 was reached [for clone 6II (Δ*lysR1*) 24 h, all other clones 16 h]. Total RNA was isolated with the UltraClean^®^ Microbial RNA Isolation Kit (MO BIO Laboratories, Inc., Carlsbad, CA, United States) according to manufacturer’s instructions with the exception that only 1 ml cell culture was harvested instead of the recommended 2 ml. Subsequently, genomic DNA was removed by using the RTS DNase^TM^ Kit (MO BIO Laboratories, Inc.) following the provided protocol, but with the modification that after half an hour an additional microliter RTS DNase was added. The reaction was incubated at 37°C for further 30 min followed by RTS DNA removal using 10 μl instead of 5 μl RTS DNase removal resins. One thousand two hundred microgram isolated RNA was used to synthesize cDNA with Invitrogen’s SuperScript^®^ VILO^TM^ cDNA Synthesis Kit (Life Technologies^TM^, Darmstadt, Germany), according to manufacturer’s instructions. The generated cDNA was used to examine the fold change of RubisCO form I (*cbbL*) and form II (*cbbM*) structural genes during expression in above mentioned transposon and double transposon clones relative to the intact version 71C2II. The expression data were normalized to the transcripts of three different genes, namely (i) the chloramphenicol-acetyltransferase (*cat*) gene, which is encoded on the fosmid vector and reflects its copy number, (ii) the RNA polymerase sigma factor *rpoD*, which is a housekeeping gene, and (iii) the 16S rRNA encoding gene. For this purpose, cDNA was diluted 1–10 and the cDNA that was derived from transcripts was used as a template for the amplification of *cbbL* and *cbbM* genes as well as the three different housekeeping genes. The SYBR^®^ Select Master Mix, CFX (Applied Biosystems^®^ by Life Technologies^TM^) and the following primer pairs were used: for (i) *cbbL* – cbbL_810F and cbbL_1115R, for (ii) *cbbM* – cbbM_647F and cbbM_976R, for (iii) *cat* – ChlR_821F and ChlR_1104R, for (iv) *rpoD* – rpoD_416F and rpoD_720R, and for (v) the 16S rRNA gene – 16S_280F and 16S_564R (for details on primer characteristics see **Table 2**). The qRT-PCR on the MJ Mini^TM^ Gradient Thermal Cycler (Bio-Rad, Hercules, CA, United States) was performed under the following conditions: 95°C for 2 min followed by 40 cycles of 98°C for 15 s, 51°C for 20 s, and 72°C for 30 s. Each run contains, next to the samples, various controls like (i) the non-template controls, (ii) the no reverse transcriptase control as well as (iii) an inter run calibrator to ensure comparability between different runs, i.e., one reaction from the previous plate was repeated on the new plate. At least two biological and three technical replicates were measured and used to calculate fold changes (2^-ΔΔCt^). Technical replicates were arithmetically averaged and resulting mean values were used to calculate an overall mean. Errors were calculated with the Gaussian propagation of error. Standard derivations of technical replicates were entered into the equation and thus propagated forward. Significant differences were calculated from log transformed values using an unpaired *t*-test with equal variance and two-tailed distribution.

**Table 2:** Primers used in this study.

Primer description	Sequence 5′-3′	T_annaeling_ [°C]	Product length [bp]
**Quantitative reverse transcriptase PCR**			
cbbL_810F 5′	AGGTCTTGCGAACTACTGTC	51	306
cbbL_1115R	CCAGAAGCAACTGGCATAAC		
cbbM_647F	TCTGCACGGTAGCACATTTC	51	330
cbbM_976R	ATTTGACGGTCCTGCTGTTG		
ChlR_821F	TAAGCATTCTGCCGACATGG	51	284
ChlR_1104R	CGATTTCCGGCAGTTTCTAC		
rpoD_416F	GATCAACGACATGGGCATTC	51	305
rpoD_720R	CGTACTGTTCCAGCAGATAG		
16S_280F	GGTCGCTTCTCTTTGTATGC	51	285
16S_564R	CCTTACCTGGTCTTGACATC		
cbbO-m_700F	ACCTCATCGCCATAATGCTC	51	223
cbbO-m_922R	TTGCCGTCATGTTACTGGTC		
cbbQ-m_2828F	GTGGCGGCATACACCATTAG	51	147
cbbQ-m_2974R	AGTCGAAGCGCACATCTTAC		
cbbQ-1_9714F	GGTAGGTCGCTTCCTAATCG	51	199
cbbQ-1_9912R	GTGCGCTTCTACCAACTCAC		
cbbO-1_12263F	CGCATGCCATCAATGGTATC	51	263
cbbO-1_12525R	GTCAATATCCGCTGGTTCAC		
**Protein expression**			
CbbLF_NheI	GCTAGCACCATGGCTAAGACTTATAAC	50^1^/60^2^	1421
CbbLR_BamHI	GGATCCGCCTTATGCTTAACATCTAGCTTAT		
CbbMF_NheI	GCTAGCATGGATCAGTCGAATCGTTATG	56^1^/62^2^	1386
CbbMR_BamHI	GGATCCGCTTTGTGTACGCCCAACTTCTC		
LysR1F_NheI	GCTAGCATGCλCTTACATATAACCGCCCAGC	62^1^/67^2^	932
LysR1R_BamHI	GGATCCGAGCGCGTGTCCGACATGG		
LysR2F_NheI	GCTAGCATGCCTGλAATTTCCATCC	51^1^/61^2^	950
LysR2R_BamHI	GGATCCGAGCGAAATTGGTTAAACG		
**Preparation of Cy3-labeled DNA fragments**			
ncrQm-M_3427F	AGCCGCTTCATAAAGTTC	56.3	371
ncrQm-M_3797R	CAGATGCAGACACAATCTAC		
ncrM-R2_4993F	TGCAGCAACTTCTAAGTAACC	62.2	369
ncrM-R2_5361R	ACAGGCGTTTAACCAATTTCG		
ncrR2 L_7191F	CTGGGCGGTTATATGTAAG	57.7	275
ncrR2-L_7465R	TACACCGGCGTTATAAGTC		
ncrS-Q1_9151F	AACCACGTTCGTTTGATTG	57.5	331
ncrS-Q1_9531R	TGGCTCGTCTTTAATAAGG		

^1^*For the first 5 cycles/^2^ for 25 additional cycles; T_*annaeling*_, annealing temperature*.

### Polar Effects

Polar effects were investigated to determine whether transposon insertions have an impact on transcript abundances of genes located downstream of an insertion site. Therefore, transcript abundances of genes located downstream of *cbbM* and *cbbL* were measured for Δ*cbbM* (22II) and for Δ*cbbL* (24II), and compared with the transcript abundances in the intact version 71C2II. Investigated genes were (i) *cbbO-m* and (ii) *cbbQ-m* in Δ*cbbM* and (iii) *cbbQ-1* and (iv) *cbbO-1* in Δ*cbbL*. The cDNA used as template was the same as that isolated before for qRT-PCR of *cbbL* and *cbbM*. The qRT-PCR conditions were the same as mentioned above for the amplification of *cbbL* and *cbbM*, but with different primers: (i) *cbbO-m* – cbbO-m_700F and cbbO-m_922R, for (ii) *cbbQ-m* – cbbQ-m_2828F and cbbQ-m_2974R, for (iii) *cbbQ-1* – cbbQ-1_9714F and cbbQ-1_9912R, and for (iv) *cbbO-1 –* cbbO-1_11263F and cbbO-1_12525R (for details on primer characteristics see **Table 2**). Three biological and three technical replicates were measured and used to calculate fold changes (2^-ΔΔCt^). Statistics were calculated in the same way as has been described for qRT-PCR data of *cbbL* and *cbbM.*

### Overexpression and Protein Purification

The four genes encoding CbbL, CbbM, LysR1, and LysR2 were cloned in the expression vector pet21a (Novagen/Merck, Darmstadt, Germany). The coding regions of the targeted genes were amplified from the fosmid DNA of the metagenome derived clone 71C2, whereby restriction sites for NheI and BamHI were inserted using following primer pairs: (i) for *cbbL* – CbbLF_NheI and CbbLR_BamHI, (ii) for *cbbM* – cbbMF_NheI and cbbMR_BamH1, (iii) for *lysR1* – LysR1F_NheI and LysR1R_BamHI, and (iv) for *lysR2* – LysR2F_NheI 5′- and LysR2R_BamHI (primer sequences, annealing temperatures and product length are listed in **Table 2**). Amplification was done with the Pfu DNA Polymerase (Thermo Fisher Scientific, Waltham, MA, United States), following manufacturer’s instructions. The amplified fragments were ligated in the pet21a expression vector (Novagen/Merck) using the previously inserted restriction sites (NheI and BamHI). This vector has a His-tag coding sequence for the C-terminus of the cloned protein. The constructed plasmids (i) pet21a::*cbbL*, (ii) pet21a::*cbbM*, (iii) pet21a::*lysR1*, and (iv) pet21a::*lysR2* were transformed into *E. coli* Rosetta-gami2 host strains. Verified clones were cultured at 17°C (CbbM) or at 22°C (CbbL, LysR1, and LysR2) in 200 ml LB supplemented with ampicillin (100 μg ml^-1^), tetracycline (10 μg ml^-1^), and chloramphenicol (12.5 μg ml^-1^) to an optical density (λ = 600 nm) of 0.7–0.8. IPTG was added to a final concentration of 0.1 mM for CbbL, 1 mM for CbbM, 1 mM for LysR1, and 0.1 mM for LysR2. The cultures were then grown over night at 17°C (CbbM) or at 22°C (CbbL, LysR1, and LysR2). Cells were harvested by centrifugation (7,600 × *g*, 8 min, and 8°C) and washed twice with 1x PBS buffer (137 mM NaCl, 2.7 mM KCl, 10 mM Na_2_HPO_4_, and 2 mM KH_2_PO_4_, pH = 7.4). Cell pellets were stored at -20°C until proceeding with His-tag purification using Ni-NTA agarose (Qiagen, Hilden, Germany) as described in protocol 14 of TheQia*expressionist* (Qiagen, 2003), with some modifications: Initially cell pellets were resuspended in 2 ml lysis buffer (containing 10 mM imidazole). Each lysate was passed through the French press in order to disrupt cells. Cellular debris were removed by centrifugation (19,580 × *g*, 20 min, and 4°C) and supernatant volumes were brought to 20 ml with lysis buffer. Afterward 2 ml Ni-NTA agarose was added to the diluted lysates, which were incubated for 2 h on ice (shaking). After pelleting (1,000 × *g*, 30 s, and 8°C) Ni-NTA resins were washed twice with washing buffer (containing 20 mM imidazole). The protein was eluted from the column with five volumes of elution buffer (containing 250 mM imidazole). The concentration of the total purified protein was measured by performing the Bradford assay as described previously (Bradford and Williams, 1976) using bovine serum albumin as a standard. The proteins were further analyzed by SDS polyacrylamide gel electrophoresis using 12% (w/v) gels and Western-immunoblotting using 6-His-specific antibodies (see Supplementary Figure 1).

### Preparation of Cy3-Labeled DNA Fragments

In preparation for the mobility shift assay four non-coding regions located within the metagenome derived RubisCO gene cluster were Cy3-labeled, namely the non-coding regions between: (i) *cbbQ-m* and *cbbM*, (ii) *cbbM* and *lysR2*, (iii) *lysR1* and *cbbL* as well as (iv) *cbbS* and *cbbQ-1*. Labeling was done during amplification using Cy3-labeled dCTP’s (1 mM, GE Healthcare, Little Chalfont, United Kingdom), a mixture of dATP, dTTP, and dGTP (2 mM), the Phusion DNA Polymerase (Thermo Fisher Scientific) and the following primer pairs: (i) ncrQm-M_3427F and ncrQm-M_3797R for the non-coding region between *cbbQ-m* and *cbbM*, (ii) ncrM-R2_4993F and ncrM-R2_5361R for the non-coding region between *cbbM* and *lysR2*, (iii) ncrR1-L_7191F and ncrR1-L_7465R for the non-coding region between *lysR1* and *cbbL*, and (iv) ncrS-Q1_9151F and ncrS-Q1_9531R for the non-coding region between *cbbS* and *cbbQ-1* (see **Table 2** for primer sequences). PCR conditions were: Denaturation at 98°C for 10 s, primer annealing for 30 s at appropriated annealing temperatures (see **Table 2**), and elongation at 72°C for 12 s (32 cycles).

### Electrophoretic Mobility Shift Assay (EMSA)

The mobility shift assay was based on a previously published protocol (Charoenpanich et al., 2013) but with modifications: Purified His_6_-CbbL, His_6_-CbbM, His_6_-LysR1, and His_6_-LysR2 were investigated for the ability to bind at the four non-coding regions amplified from the metagenome derived RubisCO gene cluster. For this purpose, proteins were tested (i) individually but also (ii) pairwise in combination with each other. The protein concentrations used for approaches with individual proteins ranged from 0 to 1,000 ng per 30 μl reaction mixture. For approaches with two different proteins up to 2,000 ng total protein per 30 μl were used in one reaction, which corresponds to a maximum of 1,000 ng of each protein and thus ensures the comparability with the single protein approaches. Regardless of whether one or two proteins were used for the assay, protein(s) was/were firstly incubated with a total of 200 ng salmon sperm DNA for 5 min at room temperature in binding buffer (50 mM Tris-HCl, 250 mM KCl, pH = 8.5) to prevent unspecific DNA shifts. After this Cy3-labeled DNA fragments were added (200 ng per 30 μl reaction) and reaction mixture was incubated at room temperature in the dark for further 20 min. Subsequently 5 μl loading dye (20% TBE buffer and 80% glycerol) was added and samples were loaded on a 5% TBE-polyacrylamide gel. Following electrophoresis at 50 mV for 3 h in cooled TBE-buffer, gels were visualized on a VersaDoc^TM^ MP4000 (Bio-Rad) at 550 nm and an exposure time of 300 ms.

### Computational Analyses

Distinct regulatory features were predicted for the DNA of the metagenome derived RubisCO gene cluster using different online tools. Promoter regions were predicted for all non-coding regions with the SoftBerry program BProm (Solovyev and Salamov, 2011). We also searched for inverted repeats, which are putatively able to fold into stem-loop structures using Emboss Palindrome (Rice et al., 2000), with a minimum length for repeats of 8 nt and a maximum gap between repeated elements of 100 nt. With respect to the formation of stem-loop structures, inverted repeats with loops less than three bases were not taken into account, because they are thought to be sterically impossible and thus are believed not to be formed (Bon and Orland, 2011). LysR binding sites were identified manually by searching for the typical LysR binding motif TnA-n7/8-AnT which has been identified in other RubisCO harboring organisms before (van Keulen et al., 2003; Maddocks and Oyston, 2008 and references therein).

## Results

To understand the processes involved in expression of a fully active RubisCO form I (CbbLS) and form II (CbbM) enzyme, we constructed two double mutant libraries using a 13 kb metagenomic fragment encoding the RubisCO gene cluster. In one case, the Δ*cbbM* fragment of transposon clone 22II, and in the other case, the Δ*cbbL* fragment of transposon clone 24II provided the base for the second mutant library. These double mutants were used to study how gene deletions influence *cbbL* and *cbbM* transcription and respective enzyme activities. We also searched through the metagenomic DNA sequence *in silico* for putative LysR binding sites, promoter regions or structures capable of forming stem-loops – possibly affecting transcription – and determined experimentally whether RubisCO and LysR proteins and protein combinations bind to non-coding regions in the metagenomic fragment.

### *cbbL* and *cbbM* Transcription after Gene Deletions

**FIGURE 1:**
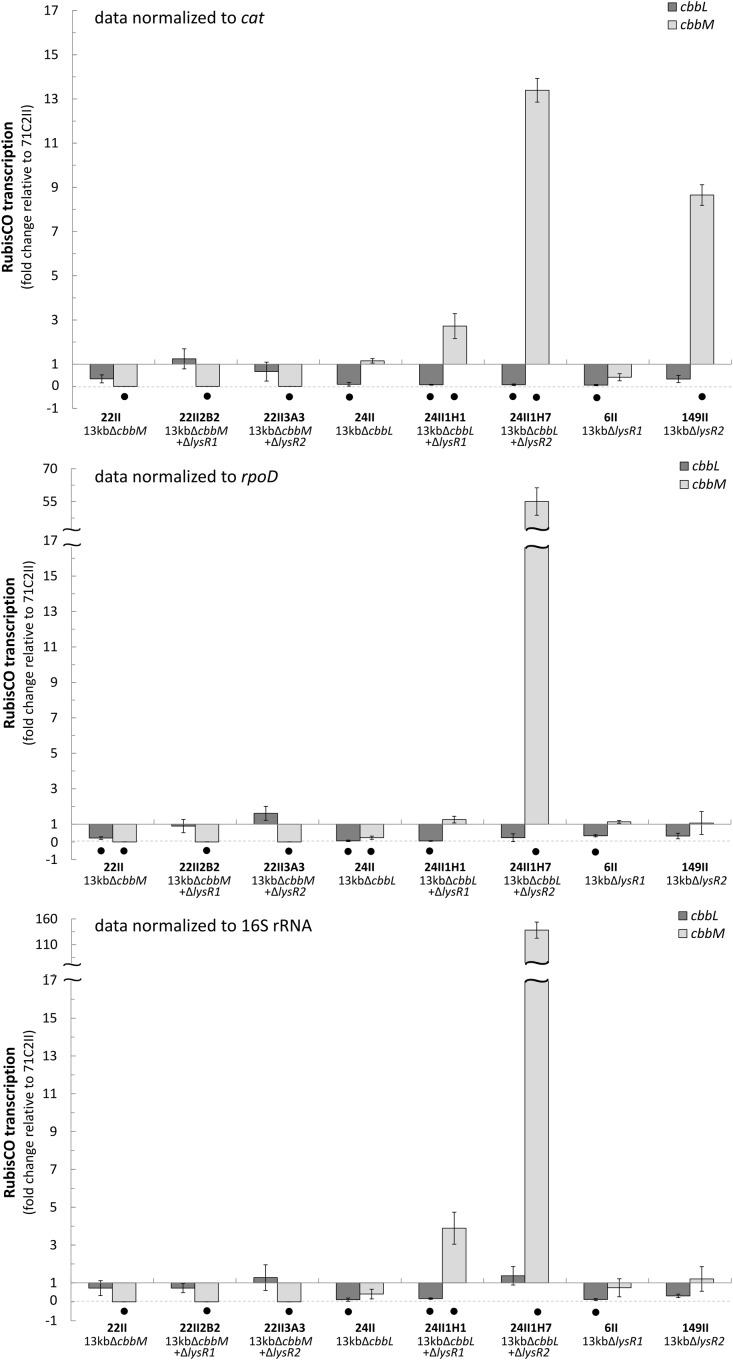
Fold change of *cbbL* and *cbbM* transcription. Fold change (2^-^^Δ^ ^ΔCt^) of *cbbL* (light-gray) and *cbbM* (dark-gray) transcripts expressed from selected transposon and double transposon clones normalized to the *cat* (chloramphenicol acetyltransferase), *rpoD*, and 16S rRNA genes. All data is relative to the 13 kb metagenomic fragment (clone 71C2II) encoding the RubisCO gene cluster (*cbbO-mQ-mM lysR*2 *lysR*1 *cbbLSQ1O1*). Bars and error bars indicate mean values and +/– standard error. Black dots denote significantly different values (*p*-value ≤ 0.05).

*cbbL* and *cbbM* transcription abundances were tested for eight mutants and normalized to three different reference genes (*cat*, *rpoD*, and 16S rRNA) (**Figure 1**). Generally, transcript levels of *cbbL* in Δ*cbbM* and of *cbbM* in Δ*cbbL* remained unchanged relative to the undeleted metagenomic fragment (71C2II). *cbbL* gene transcription was only significantly downregulated in Δ*lysR1* if *cbbM* was expressed, since no changes in *cbbL* transcript levels were observed in Δ*lysR1* Δ*cbbM*. In contrast, *cbbM* gene transcription was significantly upregulated in Δ*cbbL* Δ*lysR1* double transposon clone 24II1H1 (3-fold) and in Δ*cbbL* Δ*lysR2* double transposon clone 24II1H7 (15-fold).

### *cbbL* and *cbbM* Activity after Gene Deletions

**FIGURE 2:**
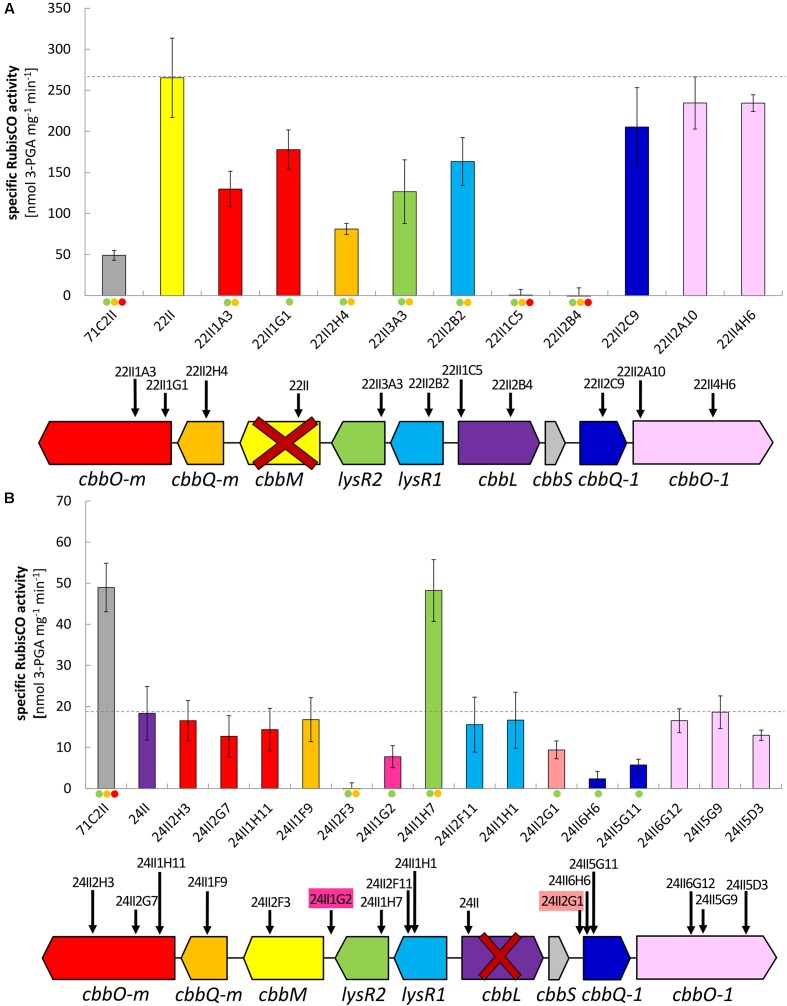
Specific RubisCO activities and insertion positions of tested double transposon clones. Specific RubisCO activities and schematic gene arrangement of **(A)** double transposon clones constructed on the basis of the Δ*cbbM* transposon clone 22II and **(B)** double transposon clones constructed on the basis of the Δ*cbbL* transposon clone 24II. Identified open reading frames (ORFs) are indicated as arrows in the direction of transcription. Insertion sites are denoted by vertical black arrows and transposon clone numbers. Genes are color coded according to the bars indicating corresponding RubisCO activity. Gene abbreviations are as follows: *cbbO-m* – von Willebrand factor type A; *cbbQ-m* – ATPase AAA-type; *cbbM* – ribulose-1,5-bisphosphate carboxylase/oxygenase large subunit, form II; *lysR2* – transcriptional regulator, LysR family; *lysR1* – transcriptional regulator, LysR family; *cbbL* – ribulose-1,5-bisphosphate carboxylase/oxygenase large subunit, form I; *cbbS* – ribulose-1,5-bisphosphate carboxylase small subunit; *cbbQ-1* – ATPase, AAA-type; *cbbO-1* – von Willebrand factor. Bars and error bars indicate mean values and +/– standard error. The level of significant differences is denoted by dots where green is ≤0.05, yellow is ≤0.01, and red is ≤0.001.

All RubisCO activities of clones from the double mutant libraries where either Δ*cbbM* (22II) or Δ*cbbL* (24II) was used for the construction of the double mutants can be viewed in **Figures 2A,B**, respectively. Total RubisCO activity of the undeleted 13 kb fragment (71C2II) increased considerably in Δ*cbbM* (22II) (5-fold). Additional deletions in *lysR1*, *lysR2*, *cbbQ-m*, and *cbbO-m* resulted in a significant decrease of RubisCO activity.

Total RubisCO activity of the undeleted 13 kb fragment (71C2II) was significantly reduced in Δ*cbbL* (24II). When additionally deleting *lysR2* (24II1H7), the RubisCO activity increased (3.5-fold), restoring the original activity of clone 71C2II. In four of the tested double mutant Δ*cbbL* clones the RubisCO activity was considerably reduced. These were clone 24II1G2, where parts of the intergenic region of *cbbM* and *lysR2* were deleted, clone 24II2G1, where parts of the non-coding region between *cbbS* and *cbbQ-1* were deleted, and the two Δ*cbbL* Δ*cbbQ-1* clones (24II6H6 and 24II5G11). As expected, no RubisCO activity was measured for Δ*cbbL* Δ*cbbM.*

### Putative Promoters, LysR Binding Sites and Stem-Loop Forming Structures

**FIGURE 3:**
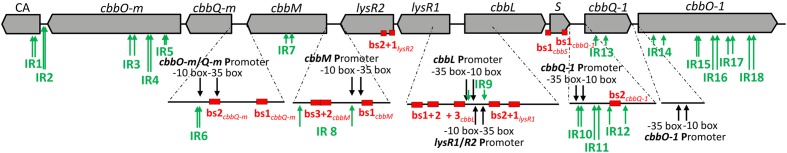
Regulatory features predicted for the DNA of the metagenome derived RubisCO gene cluster. ORFs are displayed as gray arrows in the direction of transcription. The same gene abbreviations as specified in **Figure 2** are used. Predicted promoters are indicated by black arrows, inverted repeats (IR) are denoted by green arrows and putative LysR binding sites (bs) are represented by red boxes.

We searched the intergenic regions of our metagenomic fragment for structures which encode putative promoter regions that provide potential LysR binding sites or that may form stem-loops (**Figure 3**). We found 6 putative promoter binding sites, 15 putative LysR binding sites, and 18 putative stem-loop forming structures (for exact positions on the metagenomic fragment see Supplementary Figure 2).

**FIGURE 4:**
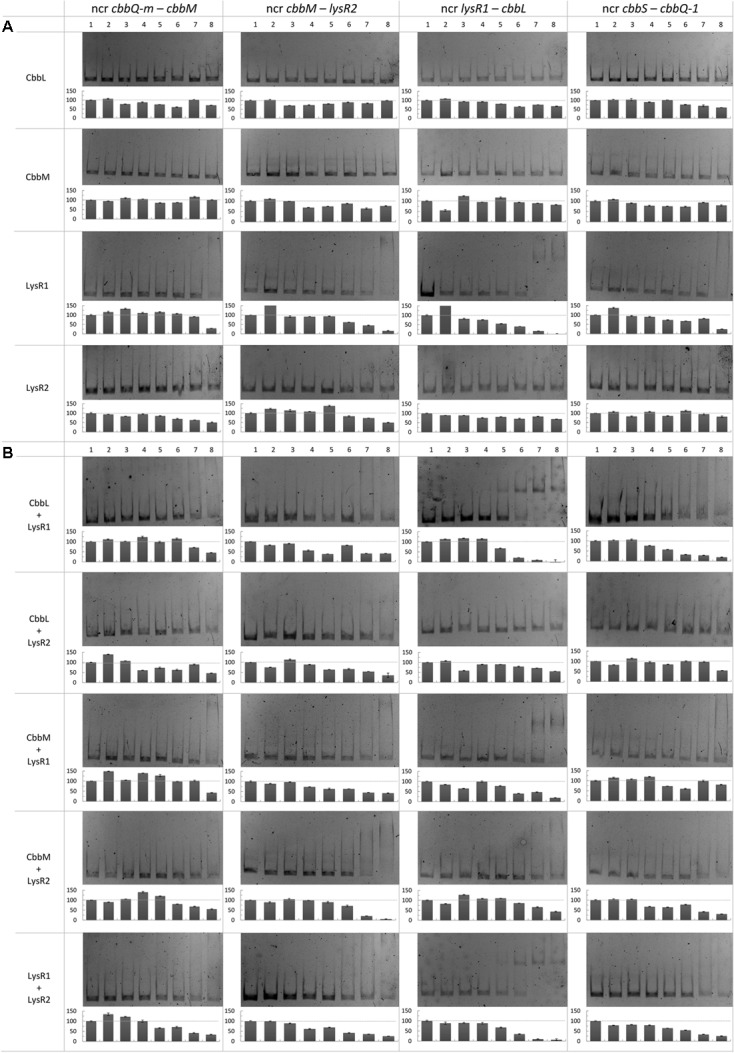
Binding of CbbL, CbbM, LysR1, and LysR2 to four different non-coding regions of the metagenome derived RubisCO gene cluster. Mobility shift assays with CbbL, CbbM, LysR1, and LysR2 individually **(A)** as well as in **(B)** combination are shown. Corresponding semi-quantitative data is depicted below each gel where the *y*-axis denotes intensity in percent relative to the unshifted band in lane 1. DNA fragments contained the non-coding regions between *cbbQ-m* and *cbbM*, *cbbM* and *lysR2*, *lysR1* and *cbbL*, and *cbbS* and *cbbQ-1*. The protein concentrations used for approaches with individual proteins, given per 30 μl reaction mixture: 0 ng (1), 10 ng (2), 25 ng (3), 50 ng (4), 100 ng (5), 250 ng (6), 500 ng (7), and 1000 ng (8). For approaches with two different proteins tested in one reaction the following protein concentrations per 30 μl reaction mixture were used: 0 ng (1), 25 ng (2), 50 ng (3), 100 ng (4), 250 ng (5), 500 ng (6), 1000 ng (7) and 2000 ng (8). Gene abbreviations are the same as described in **Figure 2**. Other abbreviation: ncr, non-coding region.

We also performed mobility shift assays to test whether LysR1, LysR2, CbbL, and CbbM or a combination of these proteins bind to the non-coding regions *cbbQ-m* and *cbbM*, *cbbM* and *lysR2*, *lysR1* and *cbbL* or *cbbS* and *cbbQ-1* (**Figure 4**). LysR1 binds to all tested non-coding regions (**Figure 4A**). Its binding ability is enhanced for the intergenic region *lysR1* and *cbbL* if CbbL or LysR2 are additionally present (**Figure 4B**). LysR2 alone appears to only bind to two non-coding regions: between *cbbQ-m* and *cbbM* and between *cbbM* and *lysR2* (**Figure 4A**). CbbM addition intensifies the binding ability to the *cbbM* and *lysR2* intergenic region (**Figure 4B**). The presence of CbbM also enables LysR2 to bind to two further non-coding regions, namely *lysR1* and *cbbL* as well as *cbbS* and *cbbQ-1* (**Figure 4B**). Other protein combinations likely reflect binding of one of the proteins alone.

## Discussion

### Possible CbbL Expression and Regulation

In the intergenic region of *lysR1* and *cbbL* two promoters were predicted: (i) one could be for *cbbL* transcription (with the -10 box ‘AGGAATCAT’ at position 7,271 bp and the -35 box ‘TTGATA’ at position 7,250 bp) and (ii) the other for *lysR1/lysR2* transcription with the -10 box at position 7,275 bp ‘ATCATATAC’ and with the -35 box at position 7,302 bp ‘TAACAA’ (Supplementary Figure 2). This is in line with previous predicted functions for the non-coding region between *lysR* and *cbbL* in other organisms, where promoters for both directions were identified (Kusano and Sugawara, 1993; Wei et al., 2004). Additionally, three and two putative LTTR binding sites upstream of the putative *cbbL* and *lysR1/lysR2* promoters, respectively, were recognized (**Figure 3**). These sites may be involved in LysR1 and/or LysR2 regulated *cbbL* transcription as well as autoregulation of their own transcription, as has been commonly demonstrated for enzymes of the LysR family (Schell, 1993; Maddocks and Oyston, 2008). The mobility shift assay verified that binding sites are located in this non-coding region (**Figure 4**). Here, DNA binding of LysR1 is intensified by the presence of LysR2 or CbbL (**Figure 4B**). LysR2 is also capable of binding to this region, but only when CbbM proteins are available. Promiscuous heterotypic interactions between different LTTRs in *E. coli* have been shown before, but the relevance of such cross-interactions remains unknown (Knapp and Hu, 2010). However, since our experiment showed that LysR1’s DNA binding ability is increased by LysR2, one may conclude that LysR1 and LysR2 are also able to cross-interact and form heteromultimers with its non-cognate partner. The heteromultimer (LysR1+LysR2) may cause different regulatory effects relative to the homomultimers (LysR1+LysR1 or LysR2+LysR2). The role that CbbL and CbbM play for intensified LysR binding currently remains unclear but may be related to DNA or RNA stability. Mobility shift assays with RNA and the large RubisCO subunit of *Chlamydomonas reinhardtii* demonstrated CbbL’s ability to bind to RNA in a sequence-independent manner under certain conditions (Yosef et al., 2004).

**FIGURE 5:**
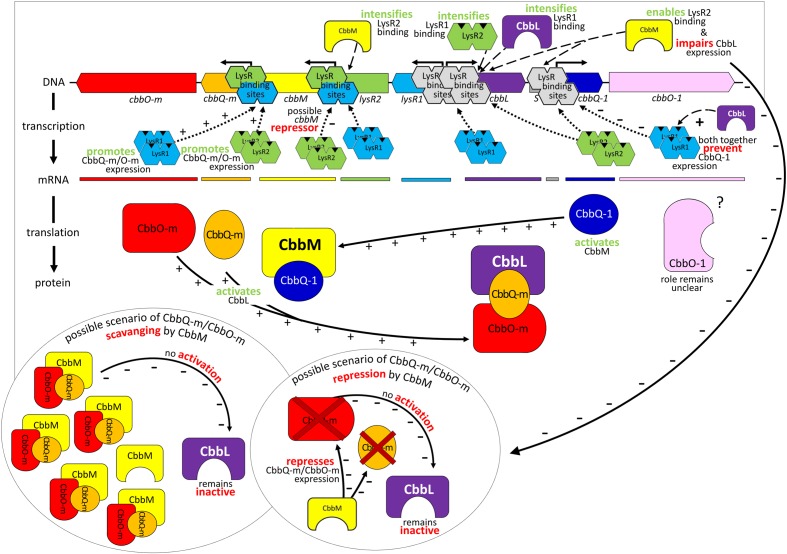
Model of gene regulation and possible protein interaction. Relevant transcriptional and post-translational regulatory processes hypothesized for the 13 kb comprising metagenome derived RubisCO gene cluster. Identified genes are indicated as arrows in the direction of transcription and marked in the same color as the corresponding mRNA and resulting gene products. Abbreviations used are the same as described in **Figure 2**.

Although *cbbL* transcription levels remained unchanged in Δ*cbbM* Δ*lysR1* and Δ*cbbM* Δ*lysR2* (**Figure 1**), these clones exhibited reduced CbbL activity (**Figure 2A**). This discrepancy may be explained if LysR proteins also act on the transcription of genes encoding proteins, which influence CbbL activity. Likely candidates encoded on this metagenomic fragment are CbbO-m, CbbQ-m, CbbQ-1, and CbbO-1, previously shown to be involved in post-translational activation of RubisCO enzymes (Tsai et al., 2015). Indeed LysR1 proteins are demonstrated to bind upstream of *cbbQ-m/cbbO-m* and *cbbQ-1/cbbO-1* regions (**Figure 4A**). LysR2 can also bind upstream of *cbbQ-m/cbbO-m* and if CbbM is present can bind upstream of *cbbQ-1/cbbO-1*, too (**Figures 4A,B**, respectively). While the deletion of *cbbQ-m* and *cbbO-m* in Δ*cbbM* illustrated a RubisCO activity loss, CbbQ-1 and CbbO-1 did not have an effect on CbbL activity (**Figure 2A**). Based on the available information, we posit that CbbQ-m/CbbO-m activates CbbL and that LysR1 and LysR2 proteins promote CbbQ-m/CbbO-m expression (compare model in **Figure 5**).

Intriguingly, CbbM also appears to play a role for *cbbL* expression, which has not been observed in any other study before. The deletion of *cbbM* leads to a 5-fold RubisCO activity increase (**Figure 2A**) indicative of CbbM’s repressive nature for *cbbL* expression. However, in Δ*cbbM cbbL* transcript levels are not elevated (**Figure 1**). Possible scenarios include that CbbM scavenges post-translational activators (CbbQ-m/CbbO-m), which are then not available for CbbL activation or that CbbM is involved in repressing CbbQ-m/CbbO-m, which may be needed for CbbL activation (**Figure 5**). For information on putative polar effects for *cbbQ-m*/*cbbO-m* transcript abundance caused by transposon insertion in the upstream neighboring *cbbM* gene see Supplementary Results and Discussion and Supplementary Figure 3.

### Possible CbbM Expression and Regulation

A putative promoter was identified upstream of *cbbM* (**Figure 3**), confirming recent results indicative of *cbbM* being transcribed alone in this fragment (Böhnke and Perner, 2015). In the intergenic region of *cbbM* and *lysR2* three putative LysR binding sites were predicted, two of which partially overlap (‘LysR bs1*_cbbM_*,’ ‘LysR bs2/3*_cbbM_*,’ see Supplementary Figure 2). In *Xanthobacter flavus* the same arrangement of a single LysR binding site followed by two overlapping binding sites, between a RubisCO structural gene and a LysR transcriptional regulator was also identified and all three sites were evidenced to be functional (van Keulen et al., 2003). In our fragment LysR binding sites upstream of *cbbM* exist, which allow binding of LysR1 and LysR2 proteins (**Figure 4A**). LysR2 binding was even intensified if CbbM was present (compare **Figure 4B**). Upregulation of the *cbbM* transcript (**Figure 1**) and increasing RubisCO activity in the double mutant Δ*cbbL* Δ*lysR2* (**Figure 2B**) strongly suggest that LysR2 acts as a repressor for *cbbM* gene expression where CbbM itself contributes to intensified LysR binding ability and thus its own transcriptional regulation (**Figure 5**). The combination of CbbL and LysR1 also appears to result in a repressive *cbbM* transcriptional regulation but single mutations in *cbbL* and *lysR1* did not cause upregulation of the *cbbM* gene (see **Figure 1**). However, despite higher *cbbM* transcript levels in Δ*cbbL* Δ*lysR1*, this clone did not demonstrate an increase in RubisCO activity (**Figure 2B**), contrasting the transcriptional data at first glance. These results can be explained though if LysR1 also controls the expression of post-translational activators, which here only seems applicable if CbbL is also present. And indeed LysR1 appears to be able to bind upstream of *cbbQ-m*/*cbbQ-m* and of *cbbQ-1*/*cbbO-1* (**Figure 4**), where also putative LysR binding sites were recognized (**Figure 3**). Of these likely post-translational activators only the deletion of *cbbQ-1* caused a CbbM activity loss and thus makes its LysR regulated role in CbbM activation under the provided conditions highly likely. We thus suggest that CbbQ-1 activates CbbM and that LysR1 proteins may prevent CbbQ-1 expression if CbbL is present. Additionally, an insertion at position 9,463 bp, i.e., 15 bp upstream of the *cbbQ-1* transcription start in the Δ*cbbL* clone 24II, resulted in significantly reduced CbbM activity (see **Figure 2B**, clone 24II2G1). Even though this insertion is located downstream of putative regulatory features, the 1,674 bp comprising <TET> insertion represents a barrier the RNA polymerase most likely cannot simply overcome, which would result in an impaired *cbbQ-1* transcription and thus no CbbQ-1 would be present. Unexpectedly, the deletion in *cbbO-1*, downstream of *cbbQ-1*, did not alter RubisCO activity (**Figure 2B**). However, the elevated number of inverted repeats (5/18 identified on the metagenomic fragment) in the *cbbO-1* gene, which are theoretically capable of forming stem-loop structures, may indicate some fine-tuned transcriptional regulation (Treangen et al., 2009 and references therein). Under the provided conditions *cbbO-1* may be downregulated and under other environmental conditions may well be important for RubisCO activation.

In the intergenic region of *cbbM* and *lysR2* a large inverted repeat flanking 86 nt (IR8, see Supplementary Figure 2) also exists. Such inverted repeats often form stem-loop structures that are important for controlling transcription initiation and termination, mRNA stabilization or genome plasticity (Treangen et al., 2009 and references therein). They also play roles in supporting DNA binding proteins in finding their binding sites (Frost et al., 1994). The IR8 in our metagenomic fragment may well represent such a signaling stem-loop structure that guides LysR to the LysR binding sites. An insertion of a tetracycline cassette in the left arm of the IR8 (clone 24II1G2), directly between the LysR binding sites ‘bs1*_cbbM_*’ and ‘bs2/3*_cbbM_*’ (see position 5,232 nt in Supplementary Figure 2) resulted in a significant RubisCO activity loss (**Figure 2B**). One explanation could be that this structure is necessary for the expression of a functional CbbM. Since this insertion also separates the putative -10 from the -35 promoter box, the RubisCO activity loss may be due to the impaired promoter region.

### Benefits and Drawbacks of Working with a Metagenomic Fragment in a Non-native System

Restricting the work to culture-depended approaches, neglects the large majority of RubisCO gene clusters from uncultured organisms. However, working with metagenomes in non-native systems holds both promise and pitfalls. The benefits of using *E. coli* as a host organism are well-known: *E. coli* has an unrivaled fast growth on inexpensive media and the genetics are very well-known, making transformations with exogenous DNA simple and straightforward (Rosano and Ceccarelli, 2014). In contrast, expression of a metagenomic fragment in a surrogate host may also entail cross-talks, inhibitions and unspecific reactions. Recombinant gene expression in *E. coli* and other surrogate hosts might be troublesome due to, e.g., unrecognized intrinsic promotors and associated factors, a diverging codon usage or problems with correct protein folding (Perner et al., 2011).

One major advantage of working with a metagenomic fragment in fosmid clones (besides gaining access to the world of the unculturables) is its relatively small size (in this case: 13 kb metagenomic fragment). The small size infers clear gene arrangements and a limited number of possible gene and/or protein interactions relative to the (hardly tangible) complexity in a native system. The same work in a cultured representative is considerably more difficult and time consuming, because genes with yet unknown functions, which are not necessarily located in the vicinity of the gene cluster under investigation, may well participate in/contribute to the gene regulation and activation of the enzyme (indirectly), as has recently been shown for *orf06* (Böhnke and Perner, 2015). Although the metagenomic approach with a defined number of genes can simplify first insights into regulatory mechanisms, it can also hinder the understanding of the mechanisms given that some vital genes/respective products cannot be expressed/synthesized as they are located on parts of the genome not present on the captured fraction of the metagenome.

To overcome such limitations the use of a host with the genomic inventory to operate the CBB cycle may be a viable option. In our case a cultured *Thiomicrospira* strain could be used and the genes under investigation deleted. However, deleting gene clusters in *T. crunogena* which are comparable to our 13 kb metagenomic fragment and expressing the latter heterologously in the *Thiomicrospira* host or alternatively constructing double mutants as we did in our metagenomic fragment (i.e., nine deletions in Δ*cbbL* and eight deletions in Δ*cbbM*) to investigate regulatory mechanisms is hardly feasible, given that *Thiomicrospira*’s genetic accessibility is not understood and thus any transformation with exogenous DNA becomes challenging. An alternative host that operates the CBB cycle and where mutations have been successfully constructed is *R. capsulatus* (*Alphaproteobacteria*) (Paoli et al., 1998; Witte et al., 2010; Dangel et al., 2014; Varaljay et al., 2016). However, this potential host encodes different types of RubisCOs and has other RubisCO gene cluster arrangements and likely different gene regulation mechanisms than the organism encoding our metagenomic fragment.

For future work one may consider combining studies in a genetically accessible surrogate host such as *E. coli*, naturally incapable of operating the CBB cycle, with subsequent investigations in a closely related cultured representative. Thus, the first insights of complex RubisCO regulatory mechanisms obtained through studies dealing with RubisCO gene expression in a non-native system could be used in further studies where, e.g., the role of external factors could be studied in a native system.

## Conclusion

The intense interactions between the different proteins suggest the complex, but fine-tuned nature of the RubisCO regulatory machinery. This fine-tuned regulatory machinery reflects the highly dynamic nature of hydrothermal vent environments from which this metagenomic fragment was extracted. Albeit the CBB cycle has a much higher energy requirement than other autotrophic CO_2_ fixation pathways (Berg et al., 2010), it can operate when O_2_ is present, while many enzymes of other CO_2_ fixation pathways are highly O_2_ sensitive (Berg, 2011). Given that RubisCO form I and form II have different capabilities to discriminate between CO_2_ and O_2_ (Berg, 2011) and both CO_2_ and O_2_ concentrations can be highly variable in hydrothermal vent habitats (Perner et al., 2013 and references therein), the ability to rapidly react to environmental CO_2_ and O_2_ changes may pose a benefit for local organisms with both forms of RubisCO. A quick response to increasing O_2_ levels may be the key to successfully colonizing dynamic hydrothermal environments. Having understood some of the possible interactions between the proteins encoded by our metagenomic fragment, this knowledge could now be transferred to a closely related cultured representative. Distinct experiments under different environmental conditions such as high/low CO_2_ or O_2_ concentrations could be performed and changes in the transcriptome investigated.

## Author Contributions

SB planned and performed experiments, performed computational analyses, and wrote the paper. MP designed the research project, planned experiments, and wrote the paper.

## Conflict of Interest Statement

The authors declare that the research was conducted in the absence of any commercial or financial relationships that could be construed as a potential conflict of interest.

## References

[B1] Amann R. I. Ludwig W. Schleifer K. H. (1995). Phylogenetic identification and in situ detection of individual microbial cells without cultivation. *Microbiol. Rev.* 59 143–169. 7535888 10.1128/mr.59.1.143-169.1995 PMC239358

[B2] Axler-DiPerte G. L. Miller V. L. Darwin A. J. (2006). YtxR, a conserved LysR-like regulator that induces expression of genes encoding a putative ADP-ribosyltransferase toxin homologue in *Yersinia enterocolitica*. *J. Bacteriol.* 188 8033–8043. 10.1128/JB.01159-06 16997967 PMC1698212

[B3] Berg I. A. (2011). Ecological aspects of the distribution of different autotrophic CO_2_ fixation pathways. *Appl. Environ. Microbiol.* 77 1925–1936. 10.1128/AEM.02473-10 21216907 PMC3067309

[B4] Berg I. A. Kockelkorn D. Ramos-Vera W. H. Say R. F. Zarzycki J. Hügler M. (2010). Autotrophic carbon fixation in archaea. *Nat. Rev. Microbiol.* 8 447–460. 10.1038/nrmicro2365 20453874

[B5] Bertani G. (1951). Studies on lysogenesis. I. The mode of phage liberation by lysogenic *Escherichia coli*. *J. Bacteriol.* 62 293–300. 14888646 10.1128/jb.62.3.293-300.1951 PMC386127

[B6] Böhnke S. Perner M. (2015). A function-based screen for seeking RubisCO active clones from metagenomes: novel enzymes influencing RubisCO activity. *ISME J.* 9 735–745. 10.1038/ismej.2014.163 25203835 PMC4331584

[B7] Bon M. Orland H. (2011). TT2NE: a novel algorithm to predict RNA secondary structures with pseudoknots. *Nucleic Acids Res.* 39:e93 10.1093/nar/gkr240 PMC3152363 21593129

[B8] Bradford M. M. Williams W. L. (1976). New, rapid, sensitive method for protein determination. *Fed. Proc.* 35 274–274.

[B9] Charoenpanich P. Meyer S. Becker A. McIntosh M. (2013). Temporal expression program of quorum sensing-based transcription regulation in *Sinorhizobium meliloti*. *J. Bacteriol.* 195 3224–3236. 10.1128/JB.00234-13 23687265 PMC3697639

[B10] Dangel A. W. Luther A. Tabita F. R. (2014). Amino acid residues of RegA important for interactions with the CbbR-DNA complex of *Rhodobacter sphaeroides*. *J. Bacteriol.* 196 3179–3190. 10.1128/JB.01842-14 24957624 PMC4135650

[B11] Dangel A. W. Tabita F. R. (2015). CbbR, the master regulator for microbial carbon dioxide fixation. *J. Bacteriol.* 197 3488–3498. 10.1128/JB.00442-15 26324454 PMC4621087

[B12] Dubbs J. M. Tabita F. R. (2003). Interactions of the cbbII promoter-operator region with CbbR and RegA (PrrA) regulators indicate distinct mechanisms to control expression of the two cbb operons of *Rhodobacter sphaeroides*. *J. Biol. Chem.* 278 16443–16450. 10.1074/jbc.M211267200 12601011

[B13] Dubbs J. M. Tabita F. R. (2004). Regulators of nonsulfur purple phototrophic bacteria and the interactive control of CO_2_ assimilation, nitrogen fixation, hydrogen metabolism and energy generation. *FEMS Microbiol. Rev.* 28 353–376. 10.1016/j.femsre.2004.01.002 15449608

[B14] Dubbs P. Dubbs J. M. Tabita F. R. (2004). Effector-mediated interaction of CbbRI and CbbRII regulators with target sequences in *Rhodobacter capsulatus*. *J. Bacteriol.* 186 8026–8035. 10.1128/JB.186.23.8026-8035.2004 15547275 PMC529060

[B15] Ellis R. J. (1979). Most abundant protein in the world. *Trends Biochem. Sci.* 4 241–244. 10.1016/0968-0004(79)90212-3

[B16] Field C. B. Behrenfeld M. J. Randerson J. T. Falkowski P. (1998). Primary production of the biosphere: integrating terrestrial and oceanic components. *Science* 281 237–240. 10.1126/science.281.5374.237 9657713

[B17] Frost L. S. Ippen-Ihler K. Skurray R. A. (1994). Analysis of the sequence and gene products of the transfer region of the F sex factor. *Microbiol. Rev.* 58 162–210. 7915817 10.1128/mr.58.2.162-210.1994 PMC372961

[B18] Hauser T. Popilka L. Hartl F. U. Hayer-Hartl M. (2015). Role of auxiliary proteins in Rubisco biogenesis and function. *Nat. Plants* 1:15065 10.1038/nplants.2015.65 27250005

[B19] Joshi G. S. Zianni M. Bobst C. E. Tabita F. R. (2013). Regulatory twist and synergistic role of metabolic coinducer- and response regulator-mediated CbbR-cbbI interactions in *Rhodopseudomonas palustris* CGA010. *J. Bacteriol.* 195 1381–1388. 10.1128/JB.02060-12 23292778 PMC3624528

[B20] Knapp G. S. Hu J. C. (2010). Specificity of the *E. coli* LysR-type transcriptional regulators. *PLoS ONE* 5:e15189 10.1371/journal.pone.0015189 PMC3004787 21187915

[B21] Kusano T. Sugawara K. (1993). Specific binding of *Thiobacillus ferrooxidans* RbcR to the intergenic sequence between the *rbc* operon and the *rbcR* gene. *J. Bacteriol.* 175 1019–1025. 10.1128/jb.175.4.1019-1025.1993 8432695 PMC193014

[B22] Maddocks S. E. Oyston P. C. F. (2008). Structure and function of the LysR-type transcriptional regulator (LTTR) family proteins. *Microbiology* 154 3609–3623. 10.1099/mic.0.2008/022772-0 19047729

[B23] Mueller-Cajar O. Stotz M. Wendler P. Hartl F. U. Bracher A. Hayer-Hartl M. (2011). Structure and function of the AAA+ protein CbbX, a red-type Rubisco activase. *Nature* 479 194–199. 10.1038/nature10568 22048315

[B24] Paoli G. C. Vichivanives P. Tabita F. R. (1998). Physiological control and regulation of the *Rhodobacter capsulatus* cbb operons. *J. Bacteriol.* 180 4258–4269. 9696777 10.1128/jb.180.16.4258-4269.1998 PMC107425

[B25] Parry M. A. Keys A. J. Madgwick P. J. Carmo-Silva A. E. Andralojc P. J. (2008). Rubisco regulation: a role for inhibitors. *J. Exp. Bot.* 59 1569–1580. 10.1093/jxb/ern084 18436543

[B26] Perner M. Gonnella G. Hourdez S. Böhnke S. Kurtz S. Girguis P. (2013). In situ chemistry and microbial community compositions in five deep-sea hydrothermal fluid samples from Irina II in the Logatchev field. *Environ. Microbiol.* 15 1551–1560. 10.1111/1462-2920.12038 23171403

[B27] Perner M. Ilmberger N. Köhler H. U. Chow J. Streit W. R. (2011). “Emerging fields in functional metagenomics and its industrial relevance: overcoming limitations and redirecting the search for novel biocatalysts,” in *Handbook of Moleculare Microbial Ecology II: Metagenomics in Different Habitats*, ed. F. J. de Bruijn (Hoboken, NJ: Blackwell), 484–485.

[B28] Qiagen (2003). *TheQiaexpressionistTM, A Handbook for High-Level Expression and Purifiction of 6xHis-Tagged Protein*, 5th Edn. Hilden: Qiagen.

[B29] Raven J. A. (2009). Contributions of anoxygenic and oxygenic phototrophy and chemolithotrophy to carbon and oxygen fluxes in aquatic environments. *Aquat. Microb. Ecol.* 56 177–192. 10.3354/ame01315

[B30] Raven J. A. (2013). Rubisco: still the most abundant protein of Earth? *New Phytol.* 198 1–3. 10.1111/nph.12197 23432200

[B31] Rice P. Longden I. Bleasby A. (2000). EMBOSS: the European molecular biology open software suite. *Trends Genet.* 16 276–277. 10.1016/S0168-9525(00)02024-2 10827456

[B32] Rosano G. L. Ceccarelli E. A. (2014). Recombinant protein expression in *Escherichia coli*: advances and challenges. *Front. Microbiol.* 5:172 10.3389/fmicb.2014.00172 PMC4029002 24860555

[B33] Schell M. A. (1993). Molecular biology of the LysR family of transcriptional regulators. *Annu. Rev. Microbiol.* 47 597–626. 10.1146/annurev.mi.47.100193.003121 8257110

[B34] Solovyev V. Salamov A. (2011). “Automatic annotation of microbial genomes and metagenomic sequences,” in *Metagenomics and Its Applications in Agriculture, Biomedicine and Environmental Studies*, ed. Li R. W. (Hauppauge, NY: Nova Science Publishers), 61–78.

[B35] Tabita F. R. Hanson T. E. Li H. Satagopan S. Singh J. Chan S. (2007). Function, structure, and evolution of the RubisCO-like proteins and their RubisCO homologs. *Microbiol. Mol. Biol. Rev.* 71 576–599. 10.1128/MMBR.00015-07 18063718 PMC2168653

[B36] Treangen T. J. Abraham A. L. Touchon M. Rocha E. P. (2009). Genesis, effects and fates of repeats in prokaryotic genomes. *FEMS Microbiol. Rev.* 33 539–571. 10.1111/j.1574-6976.2009.00169.x 19396957

[B37] Tsai Y. C. Lapina M. C. Bhushan S. Mueller-Cajar O. (2015). Identification and characterization of multiple rubisco activases in chemoautotrophic bacteria. *Nat. Commun.* 6:8883 10.1038/ncomms9883 PMC4660213 26567524

[B38] van Keulen G. Ridder A. N. Dijkhuizen L. Meijer W. G. (2003). Analysis of DNA binding and transcriptional activation by the LysR-type transcriptional regulator CbbR of *Xanthobacter flavus*. *J. Bacteriol.* 185 1245–1252. 10.1128/JB.185.4.1245-1252.2003 12562794 PMC142840

[B39] Varaljay V. A. Satagopan S. North J. A. Witte B. Dourado M. N. Anantharaman K. (2016). Functional metagenomic selection of ribulose 1 5-bisphosphate carboxylase/oxygenase from uncultivated bacteria. *Environ. Microbiol.* 18 1187–1199. 10.1111/1462-2920.13138 26617072 PMC10035430

[B40] Wei X. Sayavedra-Soto L. A. Arp D. J. (2004). The transcription of the cbb operon in *Nitrosomonas europaea*. *Microbiology* 150 1869–1879. 10.1099/mic.0.26785-0 15184573

[B41] Witte B. John D. Wawrik B. Paul J. H. Dayan D. Tabita F. R. (2010). Functional prokaryotic RubisCO from an oceanic metagenomic library. *Appl. Environ. Microbiol.* 76 2997–3003. 10.1128/AEM.02661-09 20228113 PMC2863466

[B42] Yosef I. Irihimovitch V. Knopf J. A. Cohen I. Orr-Dahan I. Nahum E. (2004). RNA binding activity of the ribulose-15-bisphosphate carboxylase/oxygenase large subunit from *Chlamydomonas reinhardtii*. *J. Biol. Chem.* 279 10148–10156. 10.1074/jbc.M308602200 14679208

