# Supplementary material for: Unraveling RubisCO Form I and Form II Regulation in an Uncultured Organism from a Deep-Sea Hydrothermal Vent via Metagenomic and Mutagenesis Studies
Source: Front Microbiol. 2017 Jul 12;8:1303. doi: 10.3389/fmicb.2017.01303 (PMC5506194; doi:10.3389/fmicb.2017.01303)
Supplement: Supplementary file 1 [file Data_Sheet_1.PDF]

Supplementary Information for

Unraveling RubisCO Form I and Form II Regulation in an Uncultured Organism from a Deep-Sea Hydrothermal Vent via Metagenomic and Mutagenesis studies

Stefanie Böhnke and Mirjam Perner*

*To whom correspondence should be addressed: mirjam.perner@uni-hamburg.de

This file contains:

Supplementary Results and Discussion

Supplementary Figures 1 - 3

Supplementary Table 1

Supplementary Results and Discussion

Polar effects caused by transposon insertions. Polar effects of the *cbbM* and *cbbL* gene deletions were tested for the downstream genes *cbbO-m*/*cbbQ-m* and *cbbO-1*/*cbbQ-1*, respectively (Supplementary Figure 3). In the *cbbM* mutant *cbbQ-m* and *cbbO-m* transcription was disturbed. In Δ*cbbL* only *cbbQ-1* transcripts were significantly downregulated. However, we consider the actual polar effects on downstream genes not of significant importance for understanding the regulatory machinery behind RubisCO expression on our metagenomic fragment for the following reasons: If indeed *cbbQ-m/cbbO-m* downregulation was detrimental to RubisCO activity then one would expect (i) a loss of activity in the *cbbM* mutant relative to the undeleted fragment, and (ii) no or little change in RubisCO activity when these genes were deleted. However, neither is the case. RubisCO activity rises 5-fold when *cbbM* is knocked out and deletion of *cbbQ-m* and *cbbO-m* results in a considerable RubisCO activity loss (Figure 2A). Since deletions in the *cbbQ-m/cbbO-m* analogues *cbbQ-1* and *cbbO-1* do not result in any change of activity, CbbQ-m/CbbO-m appear to be sufficiently present to activate the RubisCO enzyme. The same principle applies for the *cbbQ-1* gene, downregulated likely as a consequence of the insertion in the *cbbL* gene.

Supplementary Figures

Supplementary Figure 1

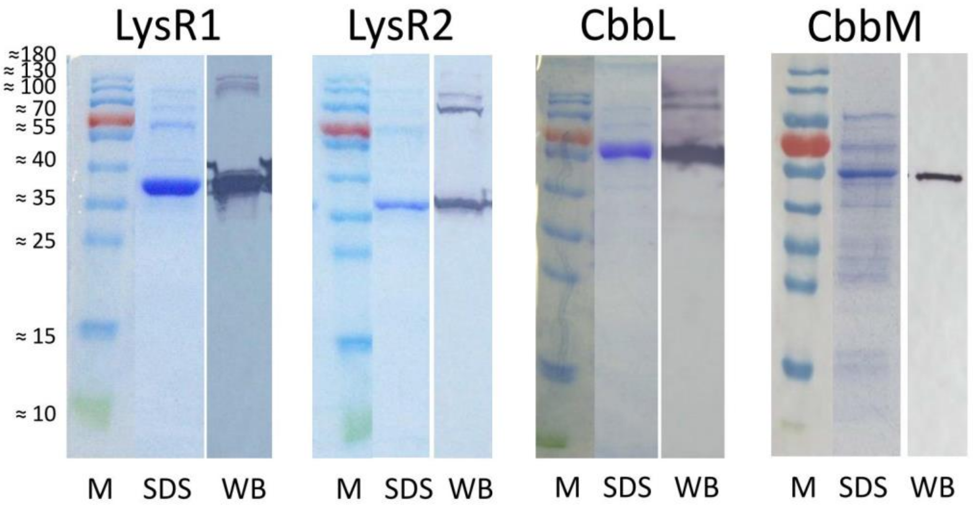

Over expression of RubisCO and RubisCO associated genes. The purified eluates of the over expressed RubisCO transcriptional regulators LysR1 and LysR2 and the RubisCO form I (CbbL) and form II (CbbM) are visualized on SDS-polyacrylamide gels (SDS) and with Western Blot (WB). The prestained protein ladder, 10-180 kDa (Thermo Fisher Scientific, Waltham, MA, USA) was used as a marker (M).

Supplementary Figure 2

```
                                                                                              IR1
  1 ATGTCACTTG AGTTGACGAG GTTGGCGATG TTTCGGTGAA CAAAAATAGT TCCTGGATCC ATCTTAACCA GCTCGTTTGC
    ---------- ---------- ----   carbonic anhydrase   ---- ---------- ---------- ----------

 81 AGGAACTCGG CTATCGGAGC AACCTATCCA GAGATATTCG GGTTTTTGTT GGTGAGATAG GGTTTCAAAA AAGTCGGGAC
    ---------- ---------- ----   carbonic anhydrase   ---- ---------- ---------- ----------

161 GAATGGCGTT GATTTCATCC ACCCAATCAC GGTTGTTTTG TAACAGCTGC TCGATTGATG TATCGCAGTC ACATTGATGG
    ---------- ---------- ----   carbonic anhydrase   ---- ---------- ---------- ----------
                                                                   IR2
241 CACATTAAGA ATCCTTTTGG AGAAGTTGTT TTGGGGATAC AAAAATGCCC TTAAATTGTA AGGGCATTTA CTTTAAAGCT
    -----

321 TCTTCAAAAT GGGAATTATA CACGTTTTTA TTGTGTGATT TTCATAAAGA CTTGAGGTAA TTTTTCTGGC AACTTGTTGA
                                ---- ---------- ----------   cbbO-m   - ---------- ----------

401 CATGGTCAAT GACGGTATAG TGGTTGTCAA AAATGGTCTC AACGTATTCA TCGGCATTCG GATCTAAGGT AATGCAGTAA
    ---------- ---------- ---------- -----     cbbO-m    ---- ---------- ---------- ----------

481 GAATAGATCC CTTTGCTTTT AAGTTCTTCG ACCGCTTTAT GGGTGTCTTG AATTAAAACC TGTGGGTCTT TTGTGTCAAT
    ---------- ---------- ---------- -----     cbbO-m    ---- ---------- ---------- ----------

561 ATCCGCCGGC TCACCATCGG TTAAAATCAG CATCAGTTTT TTCTCAGCTT GCTGTGCTTC CAGATAGTGT GCGGCATGAC
    ---------- ---------- ---------- -----     cbbO-m    ---- ---------- ---------- ----------

641 GCATGGCGGC TCCCATTCGA GTCGAATAAG AGGCTTCCAT CGCGGCAATG CGAGACTTGA CCTCATCGCC ATAATGCTCT
    ---------- ---------- ---------- -----     cbbO-m    ---- ---------- ---------- ----------

721 GAATAGCCTT TGATATGCTG ATAGCGCACT TCATGACGCG TGTCCGAGCA AAAGCCCGCA ATGGCGAACT TATCCCCTAG
    ---------- ---------- ---------- -----     cbbO-m    ---- ---------- ---------- ----------

801 CTGTTCAATG GTCCACGCAG TGATGGCAAG GGCTTCTTCA CTTAATTCCA GTAAAGTTTG GCCCGTTTCC TGATTGCGTT
    ---------- ---------- ---------- -----     cbbO-m    ---- ---------- ---------- ----------
```

```
 881 CATTCAACGA TTGTGAGGTG TCGACCAGTA ACATGACGGC AATGTTTCGG CTGTCGGTAG TATGGCTGTA GTTGATTCGA
     ---------- ---------- ---------- -----    cbbO-m    ---- ---------- ---------- ----------

 961 GGGTCCGGTG CTTGCCCGCT TTTAAAGTCA ATGACGGAAC GAATCGCGAC GTCCAAATCC AACTCTTCCC CTTCTTCCTG
     ---------- ---------- ---------- -----    cbbO-m    ---- ---------- ---------- ----------

1041 AAACCGAATG CGTTTTTTGT TTTGAGGTTT GAGGGCTTCA ATCATTTTTT TCAAGCGTTT GGCCAAACCG TCATGTTTTT
     ---------- ---------- ---------- -----    cbbO-m    ---- ---------- ---------- ----------

1121 CCATCAGGCG GTCAATTTTG TTTGAATCTC CAGAAGGGTG TAAGCGTTCA TAAACCGTCG CCCAGTCTGG ACGATAGCTT
     ---------- ---------- ---------- -----    cbbO-m    ---- ---------- ---------- ----------

1201 TCGGAGATAT AATCCCATTC GTCATAGTGG CGGGGAGGTA AGCTGTTTGC GTCGTCGACC ACTTTTTCAT CGGAGTGATA
     ---------- ---------- ---------- -----    cbbO-m    ---- ---------- ---------- ----------

1281 TTCGTTGTTA TGGAAATCTT CCGCTTCGTC ATTTTCTTCG TGATGGAACC AGATAAAGCG ATTGTCATCT CGATAGCTGA
     ---------- ---------- ---------- -----    cbbO-m    ---- ---------- ---------- ----------

1361 TTTCAGTATC GTCAAAGAAA ACGTTTGGCA AGCTGTCGGT TTTTTTGCGC GATTTGACAT AGAAGTTCGT GCCCAGTTTG
     ---------- ---------- ---------- -----    cbbO-m    ---- ---------- ---------- ----------

1441 GCCATGTCTT GTGTGGTGGA GGCGTCGCCT TTTTCAGCCA TAATGGCATT GAACTGGGTG CGGAAGTCTT CGATCAATGG
     ---------- ---------- ---------- -----    cbbO-m    ---- ---------- ---------- ----------

                                                                          IR3
1521 GTTTTCTGGG TCAAATGTTT CATCCATTAA GGCGCGAGAC AGACGGGTGG ATCGGTATCG TAAACAGGCC TGTTGGGTCG
     ---------- ---------- ---------- -----    cbbO-m    ---- ---------- ---------- ----------

1601 GATCGCACGC GCCTTTTTCC GGCACTGGGT GGAGTGCAAG AAACAAAGGT TTTAATCCGG GGAAGCGTTG AATGGCCAAT
     ---------- ---------- ---------- -----    cbbO-m    ---- ---------- ---------- ----------

1681 CTTTCCACTC GGCAGTCTTC AAAGGTGGAA ATAAAGAGCT GCATGTGAGG GGCGAAATTA TCGGCCATCA ACTTGGTTGA
     ---------- ---------- ---------- -----    cbbO-m    ---- ---------- ---------- ----------
```

```
1761 CCATTCTTTG TGCGCCATCA TATGCGCCAG CATGGCGCGA TAGCGATCCA AGCCGCTAAC ACCATTTTCA TCTTCATACA
     ---------- ---------- ---------- -----     cbbO-m    ---- ---------- ---------- ----------

1841 CATCTGGCAC GGCAATGAGT TCTTCATCCA AATACGGAAC GGGTTTACGA AGTTGATCGA ATGCGGTGGA GAAAACCGAA
     ---------- ---------- ---------- -----     cbbO-m    ---- ---------- ---------- ----------

1921 AAAGGGCGGT CACAATGCCA CATGCAGTCT TTTAACATAT CCAAATGACG TTCCACGTCT TTGAACGTTG TGCCTTGACG
     ---------- ---------- ---------- -----     cbbO-m    ---- ---------- ---------- ----------
                                                   IR4
2001 TTCACGTTGA ATGATGCTTT TGGCATCGTG CGATTCTAAG CTGAAATAGG CAATTTGCTG ATCGGGCGCA TCATTATAGT
     ---------- ---------- ---------- -----     cbbO-m    ---- ---------- ---------- ----------

2081 TACGCGCGCC ATAATCAATA AAATTACGGA TTCCGGCCAG GCTTAATTTG GAAATCAGTT GGGGCATGGA TTCCAGTAAA
     ---------- ---------- ---------- -----     cbbO-m    ---- ---------- ---------- ----------

2161 GGAATCATGC CCGGACTTTC ATACAAAGAG TGATGACCAT GAATGACGGT TTGCGTTTTG TCGACGTATT CATCAATGAT
     ---------- ---------- ---------- -----     cbbO-m    ---- ---------- ---------- ----------
                                                                                     IR5
2241 GTCTAGATAG TGCTGAAGGT CTTCCAGCGT ATCGATGCGT CGGCAGACAC TGCTGAGTGA GGCCAAAAAA GGAATTAAGG
     ---------- ---------- ---------- -----     cbbO-m    ---- ---------- ---------- ----------

2321 CTTTTTTGTT AGGGCTGCGC GCTAACTTAT AGCCGTAGTC GGCCACCATT TTCATGGTGC CTTTTCCAAT ATGAGAGGCG
     ---------- ---------- ---------- -----     cbbO-m    ---- ---------- ---------- ----------

2401 ATTTCCGGCA TGATCTCAAG GTAGACCAGA ACCGGCTCTA CGCCCATGCC TATCTTACAA AGGAAGTTGG CGCCATCAAT
     ---------- ---------- ---------- -----     cbbO-m    ---- ---------- ---------- ----------

2481 ATAGGCAGTC ACCCCTTCGT CTGAAAGTTG CTGGGCAGCC TCTTCGATAC ACGCTGGAAA AACTTCCAAC GCTTTGGGAA
     ---------- ---------- ---------- -----     cbbO-m    ---- ---------- ---------- ----------

2561 AATTACAGGT GAATTTGTCT TGATACTCAG AAACGACTTC TGTTATGACG TCTGCATTCA TAATCTAACT CATTTGATTG
     ---------- ---------- ---------- -----     cbbO-m    ---- ---------- -
```

```
2641 AATGATAAAT CGTCACCGCT CATGTTTGGG CGGTGGCTTA GCAAAAATAA CCAGGCCTGG CTGTTTTTAG CCAAAAATCA
                                                                               ---  cbbQ-m  --

2721 TTTCAATGGC GTTATCAAGG GTCTGACGAA TATCGGCATC GTCGGTAATG GGACGAACCA AAGCCATTTT ACAGGCTTCA
     ---------- ---------- ---------- -----   cbbQ-m    ---- ---------- ---------- ----------

2801 ATCGGCGCAA TACCTTGGTT AATCAGGGTG GCGGCATACA CCATTAGACG CGTTGAAATC CCTTCATCCA GCCCGTGGCC
     ---------- ---------- ---------- -----   cbbQ-m    ---- ---------- ---------- ----------

2881 TTTCAGGTTA CGAGCGGTTT CACCGATCTT AACCAGTTTT TGGGCCGTAT CAGAGTCGAC ATTCCCTTCC TTTTGTAAGA
     ---------- ---------- ---------- -----   cbbQ-m    ---- ---------- ---------- ----------

2961 TGTGCGCTTC GACTTCTGGT AGAGCATAAT CAAAGTCCAA TGCACAAAAA CGTTGTTTGG TTGATTGCTT TAAATCTTTC
     ---------- ---------- ---------- -----   cbbQ-m    ---- ---------- ---------- ----------

3041 ATCAGTGATT GATAACCCGG GTTGTATGAA ATCACTAACT GAAAATCAGG GTGGGCTTTA ATCAACTCGC CTTTTTTATC
     ---------- ---------- ---------- -----   cbbQ-m    ---- ---------- ---------- ----------

3121 TAAAGACAAT TCACGACGAT GGTCCGTTAA CGCATGAATG ACAACCATGG TGTCTTGACG TGCTTCCACA ATTTCATCTA
     ---------- ---------- ---------- -----   cbbQ-m    ---- ---------- ---------- ----------

3201 AATAACAGAT GGCACCATAA CGTGCGGCCA GCGTTAAAGG CCCATCCACC CAGCGAGTGC CGTTGGCATC CAGCAAATAA
     ---------- ---------- ---------- -----   cbbQ-m    ---- ---------- ---------- ----------

3281 CGCCCAACCA GGTCTGATGC AGTGATGTCT TCATTACACG AAACGGTAAT AATCGGTTTG CCTAATTTCC AGGCCATGTG
     ---------- ---------- ---------- -----   cbbQ-m    ---- ---------- ---------- ----------

3361 TTCGACAAAG CGGGATTTAC CACAGCCCGT CGGACCTTTC ACCATGACCG GTAAGCGAGC GGCATAAGCC GCTTCATAAA
     ---------- ---------- ---------- -----   cbbQ-m    ---- ---------- ---------- ----------

3441 GTTCAACCTC ATTGGATTGA GCATCATAAA ATGGTTCGTT TTCAATCTTG TATTGTGTAA TGTCCATATC AGTTCCCCGT
     ---------- ---------- ---------- -----   cbbQ-m    ---- ---------- -------
```

```
                                                -10 box cbbQ-m    -35 box cbbQ-m
                                                  ------ ---        ------
                                         IR6                              LysR bs2cbbQ-m
3521 AACGTTTATG GTAGGTATTT TCAAACAGAG TGTGAAAATA CCGATGAATA AATTAAAGCG ATATTAAAGT CATTTAGGTT
                                                                   -------- ------
                                                                   TnA - n7 - TnA

3601 TCTGGTTTAC CGTAAGACAG GTAAAAGAGA AAACTAAATA ACCTTGTGTT TGGCTAGGTT TTTGGTTTGG GTGTTAAAAA

                                                  LysR bs1cbbQ-m
3681 AACCCCTGCG CGAACGCAGA GGTTTCTTGG GTGTTTAAAT AAACATTCGT TAAAGAATGG CTTATTTGTG TACGCCCAAC
                                                - ---------- --              ------   cbbM   ------
                                                T nA -n7 - T nA

3761 TTCTCTCTCC AACCTGGGTA GATTGTGTCT GCATCTGCAG GGAAAGATTC GAATGCGCGA GCGAACTCTT TGTGATCTTT
     ---------- ---------- ---------- -----     cbbM    ----- ---------- ---------- ----------

3841 TGCGAATTCG ATTGGATCTG CACCTGATTT CCAGCACTCG TAAGATTGAC GAAGTGACGT TGCACCTGCC GCTGGAGAAT
     ---------- ---------- ---------- -----     cbbM    ----- ---------- ---------- ----------

3921 CGATGTGACC GTAAGAACCA CCACCAGAAG TGTTGATAAC GTTCCCGTGA CCTAGGTTTT CGAAGAAACC AGGTAGACGT
     ---------- ---------- ---------- -----     cbbM    ----- ---------- ---------- ----------

4001 AGTGCGTTCA TACCACCAGA GATGATAGGA GTCGTTGGCT TCATACCATT CCATTTCTGG TAGAAAGCTG GCCCTTGACA
     ---------- ---------- ---------- -----     cbbM    ----- ---------- ---------- ----------

4081 CTCATCACGC TCGATCATGT ATGCGATGTT CTTATCAGAA GCATCACCTT CCATTTTACC GTAACCCATT GTGCCCACGT
     ---------- ---------- ---------- -----     cbbM    ----- ---------- ---------- ----------

4161 GGATACCAGA CGCACCTTGT AGACGAGAGA TTTTAGCAAG AACGAATGCC GTGTAACCAC GTTTCGCTGA AGGCGACGTG
     ---------- ---------- ---------- -----     cbbM    ----- ---------- ---------- ----------

4241 ATCGCACCAT GACCTGCACG GTGATAATGT AAGTACTGTG AAGGATAGTT ACGACGTGCT GTCGTAATCA TACCAGGACC
     ---------- ---------- ---------- -----     cbbM    ----- ---------- ---------- ----------
      IR7
4321 ACCAACATAA CCATCAACTA GGAAAGCAAC CTGAGGCGCG TCAGCACCGA AAGTTTCTAG GATGTAATCT GCACGGTAGC
     ---------- ---------- ---------- -----     cbbM    ----- ---------- ---------- ----------
```

```
                 IR7
4401 ACATTTCATG GTGGTCATCT GCAGTGATGT TTGCTGAGAA TAACTTGGTT TCACCCGTTT CATCTTGAGC ACGCTTCATT
     ---------- ---------- ---------- -----     cbbM     ----- ---------- ---------- ----------

4481 GCGTCAGCAA CAAGCGGAAT GACTTTTTTC ATTGGGCAGA ATGTTTGGTT ACCTTGTGGC TCATCGTTTT TGATGAAATC
     ---------- ---------- ---------- -----     cbbM     ----- ---------- ---------- ----------

4561 ACCACCTAGC CAGAACTGGT ATGCTGCGTC AGCAAATGGC TCAGGACGTA GACCTAGCTT AGGCTTGATG ATTGTACCTG
     ---------- ---------- ---------- -----     cbbM     ----- ---------- ---------- ----------

4641 CGATGTAACC ACCGTTGTCT TTATCACGAC CAAGCATTTT CCACATGTTT GTGATATCAA CAGCAGGACC GTCAAATAGG
     ---------- ---------- ---------- -----     cbbM     ----- ---------- ---------- ----------

4721 TGAAGTTTAG TTTCTGGTAC CCAGAAATCA AACATTTGAA GGTTTTTAAC GTCACCCATG CCTTGGTTGT TACCAATGGC
     ---------- ---------- ---------- -----     cbbM     ----- ---------- ---------- ----------

4801 AAGAGTCAAG AAAGAAACGA TCATTGTTTT ACCGTCAAGC CCGTTACGAT CGAAAAGATC GAAAGGGTAG GCAACCTTCA
     ---------- ---------- ---------- -----     cbbM     ----- ---------- ---------- ----------

4881 TGATTCCGTT TGCTTCGTCG ATGTCATAAA CAATCGCGTC AACGCCTTTC GTGAAGTCAT CTGTTGTACA AACTTCAACG
     ---------- ---------- ---------- -----     cbbM     ----- ---------- ---------- ----------

4961 TTTGTTCCAG TTGAAGATTC CGCTGCGATG TGTGCAGCAA CTTCTAAGTA ACCGTAACCA GCAGCAGGTT CCATTGTGTA
     ---------- ---------- ---------- -----     cbbM     ----- ---------- ---------- ----------

5041 AGCAACAAGA ATGTGATTCT GACCAGCAAT CAGATCTTCT TCTTTTAGGC TTAAATCAGC ATAACGATTC GACTGATCCA
     ---------- ---------- ---------- -----     cbbM     ----- ---------- ---------- ----------
                IR8                                                  LysR bs3cbbM LysR bs2cbbM
5121 TGTTTTATAC CTCTATATAT TGGGACAACA GCTCAGGAAA GTGTGTCCAT AATTTTTGTT AAGTCATTGT AAAAACAACT
     -                                                              Tn A – n7 – T nA
                          -10 box cbbM             -35 box cbbM               T nA – n8 –
                          --------- -              ------     LysR bs1cbbM
5201 TAACCCCGAA ATTTGAATGG TCATTATAGA GGTTGTTCAG ATAACTTCAA ATCAATATTT TTGATTAAAT TGATAGACTA
     TnA                           IR8                         TnA – n7 – TnA
```

```
5281 TTATCTATTC TAGTGAGGTG CGATTTAAAT GATGCGTAAT AATCTGCAAA GTTGGGTTAG CGAAATTGGT TAAACGCCTG
                                                                ---- --   lysR2   --------

5361 TTCGCTGAGT TTTTCGCCTT CGATTTTCAG TAACTCAATG AGTTTTTCTG CCGCAATAGA CAGTACTTTG CCTTTTGGGT
     ---------- ---------- ---------- -----   lysR2   ----- ---------- ---------- ----------

5441 AAGCCAGGTA CCAATGACGA TTGATTGGAA ATCCTTTGAC ATTGAGGATG CTGATTTTTC CTTGATTAAT TTCTTCCATT
     ---------- ---------- ---------- -----   lysR2   ----- ---------- ---------- ----------

5521 AAAGTGGGAA CCGATGCAAC CGTGATTCCA AGGTTTTGTA GTAAGCCGAG TCGAATAGGT TCGTTGCCAC CCAGCACCAT
     ---------- ---------- ---------- -----   lysR2   ----- ---------- ---------- ----------

5601 TTTAATATTG GGTTGAAATT CAAATTCTCC AAAGACCTTT TCAATTTGAG CCCGAATACC TGAACCCGTT TCGCGCATTA
     ---------- ---------- ---------- -----   lysR2   ----- ---------- ---------- ----------

5681 AAAAAGGTTC ATTTACCAGG TCTTGAATTG AGAGTTGCTT TCCAACGAGA GGGTGATCAC TGTTGGCCAA AAAAGCCAAT
     ---------- ---------- ---------- -----   lysR2   ----- ---------- ---------- ----------

5761 GGGTTGACGG AGAGCTGAGA AGATTCCACA TTTAAGTCTT CTGGCGGTTG TCCGAGTAAA TAAAAGTCGT CCTTGTTATT
     ---------- ---------- ---------- -----   lysR2   ----- ---------- ---------- ----------

5841 ATTAATACGT TCGATCAAAC TTTCTTTGTT ACCAACCCGC ATGATGATGG TGACGTCTGG GTAGGATTGC GTGAAATTTT
     ---------- ---------- ---------- -----   lysR2   ----- ---------- ---------- ----------

5921 GAATGACTTT AGGCACAAAA TATTGCGCCG TTGAGATTAC CGTAAATTTG ACGCTGCCCC CAGAAAACCC CTTGAGGTGA
     ---------- ---------- ---------- -----   lysR2   ----- ---------- ---------- ----------

        LysR bs2_lysR2
6001 TTGATTTTCT GTTCAGCAAT CGACAGTTGC TGCAAGATAT TATTGGCGGA ATCGTAGAGT GCTTTACCGG CTTCTGTTAA
     ---------- ---------- ---------- -----   lysR2   ----- ---------- ---------- ----------
      TnA  - n8 - TnA

6081 ATGGGCTTTA CGACCGATTT GTTCAAATAA AGGACTTTCC AAAATTTCGG CCAGTTTCTT AACCTGCATG GAAACCGTTG
     ---------- ---------- ---------- -----   lysR2   ----- ---------- ---------- ----------
```

```
6161 GTTGAGTCAG GTGAAGCTCT TCAGCTGCTT TGGAGAAGCT GTTATGTCGG GCAATGCTTT CAAAGACTTG AAGCTGTCTT
     ---------- ---------- ---------- -----   lysR2   ----- ---------- ---------- ----------

                                      LysR bs1_lysR2
6241 AATGTGGCGT TTCGTGCCAG GATGGAAATT TTTTCAGGCA TAATGATTCC TTAGCGCGTG TCCGACATGG CCAGCAATTT
     ---------- -----   lysR2   ----- ---------- -            ---------- --   lysR1   ---------
                                        TnA - n7 - TnA

6321 GTTAATGTCG ACCGATTTCA GAAATTCCAA AAAGGCGACC GCTGGTGGCA TGAGTACCTT ATCTTGCATT TTGGCAATGT
     ---------- ---------- ---------- -----   lysR1   ----- ---------- ---------- ----------

6401 ACCAATCGCG ACTCAATGGA AACCCTTGAA CGTCAAGAAC GGTTAAGTGA TCGTATTTTG CTTCGATGCG CACCGCGTGT
     ---------- ---------- ---------- -----   lysR1   ----- ---------- ---------- ----------

6481 CGGGCAATGA CAGAAATACC CAGGCCTGCC ATAACCCCTT GCTTGATTGC TTCGGTACTG CCCAGTTCCA TGTAGGGCTG
     ---------- ---------- ---------- -----   lysR1   ----- ---------- ---------- ----------

6561 GATAGAATAC CCTTCTTCGG CAAAGCGTTC TTCTGTGGCA ATACGGATGC CGGACCCGGC TTCTCGCATT AAAAAGTTTT
     ---------- ---------- ---------- -----   lysR1   ----- ---------- ---------- ----------

6641 CATTTACAAG GTCGCTCAAT GGGATTTGTT TATGTTTTGC AAGTGGGTGA TTGGGCGGAG CGGCCACAAT CAATTCATTC
     ---------- ---------- ---------- -----   lysR1   ----- ---------- ---------- ----------

6721 TTGAAGAATG GGAAAGCTTC CATCTTTAAT TCCGGTGGTA CTCTTCCCAT AATAGAGAGG TGATATTGGT TTTGTTTAAG
     ---------- ---------- ---------- -----   lysR1   ----- ---------- ---------- ----------

6801 CTCATCTATG ATGCGTCTTC TATTAACAAC TGTTATGGAA GGAATGACAT CTGGGTATTT ATTTAAAAAT GCTTTCAAAA
     ---------- ---------- ---------- -----   lysR1   ----- ---------- ---------- ----------

                                 LysR bs1_cbbL
6881 CATAAGGAAT AAAAAAATTG GCTGGGGTTA CAACCGCTAA TTTCAGTTCC CCTTCAATTT TATTTTGATC TGTCTTAATG
     ---------- ---------- ---------- ---------- ---  lysR1   -------- ---------- ----------
                                TnA - n7 - TnA                                       TnA -
    LysR bs2_cbbL
6961 TTGGTGTTAA GGGTCTTGAG CTCTTCTAGA ATGGTGGTGC AGGTCTTAAA CATGTGTTCG CCTGCGGATG TTAAATAGAG
     ---------- ---------- ---------- -----   lysR1   ----- ---------- ---------- ----------
      N8 - TnA
```

```
7041 TTTTTTTCCG ATCACTTCGA TCAATTTAAT TTCGTTATTT TCTTCCAGAC GCTTAACTTG AACGGATACG GCAGGTTGAG
     ---------- ---------- ---------- -----     lysR1     ----- ---------- ---------- ----------

7121 AAAGGTTGAG TTCTTCGGCT GCTTTTGTGT AGCTTAAGTG CCTTGAAACA GCCTCGAAAA TCCTTATCTG CTGGGCGGTT
     ---------- ---------- ---------- -----     lysR1     ----- ---------- ---------- ----------
                                                           -35 box cbbL                -10 box cbbL
                                                 LysR bs3cbbL - -----                  ---------
7201 ATATGTAAGT TTTGCATTTG GTTTTCCTAA TTGGTAAACT CATAAATGGT TGATATATAA AGTAAATGAG AGGAATCATA
                                                                                        ------
     ----   lysR1   ---                          T nA - n8 -  TnA  IR9                -10 box lysR1
                                 -35 box lysR1
                                                                LysR bs2lysR1
                                    ------
7281 TACATTTGTT TATGAATTGT ATAACAAATA CTCAATATAT CACCAGATTA TTTTAGATAA GATACGAATC ACTGAAAGCG
     ---
                                                IR9          TnA - n7 - TnA
                LysR bs1lysR1
7361 GAAGTGTTTT TAAAACGCAA TTTAAGAATC GCTTTCTCTA ACTCTTTTCA TGATTAAACT AAAACGAAGA GGTAAACCAT
                TnA  - n8 - TnA                                                          --

7441 GGCTAAGACT TATAACGCCG GTGTAAAAGA ATACCGCGAA ACGTATTGGA TGCCAGAATA TGAGCCTAAA GACTCAGATT
     ---------- ---------- ---------- -----     cbbL      ----- ---------- ---------- ----------

7521 TTCTAGCATG TTTTAAAGTA ATCCCACAGG ATGGTGTTCC ACGTGAAGAA ATCGCAGCAG CTGTTGCGGC TGAATCTTCA
     ---------- ---------- ---------- -----     cbbL      ----- ---------- ---------- ----------

7601 ACGGGTACAT GGACAACTGT TTGGACTGAC TTGTTAACAG ATCTTGACTA TTATAAAGGT CGCGCATACA AAATTGAAGA
     ---------- ---------- ---------- -----     cbbL      ----- ---------- ---------- ----------

7681 CGTTCCTGGT GACGATGCAG CATTTTATGC TTTCATCGCA TACCCAATCG ATCTATTCGA AGAAGGTTCT GTCGTTTCTG
     ---------- ---------- ---------- -----     cbbL      ----- ---------- ---------- ----------

7761 TAATGACATC TTTAGTTGGT AACGTATTCG GATTTAAAGC ACTACGTGCT TGTCGTCTAG AAGATATCCG TTTCCCTCTA
     ---------- ---------- ---------- -----     cbbL      ----- ---------- ---------- ----------

7841 GCGTATGTGA TGACGTGTGG TGGACCACCA CACGGTATCC AGGTAGAGCG TGACAAAATG GATAAGTATG GTCGTCCAAT
     ---------- ---------- ---------- -----     cbbL      ----- ---------- ---------- ----------
```

```
7921 GCTAGGTTGT ACTATCAAGC CTAAGCTAGG TCTATCGGCT AAAAACTACG GTCGTGCTGT ATATGAGTGT CTACGTGGTG
     ---------- ---------- ---------- -----    cbbL    ----- ---------- ---------- ----------

8001 GTCTTGACTT CACGAAAGAT GATGAAAACG TTACTTCTCA GCCGTTCATG CGTTGGAGAG ATCGTTTCCT ATTCTGTCAA
     ---------- ---------- ---------- -----    cbbL    ----- ---------- ---------- ----------

8081 GATGCAATCG AAAAATCACA AGCAGAAACG GGTGAGCGTA AAGGTCACTA CCTAAACTGT ACAGCTGGTA CGCCAGAAGA
     ---------- ---------- ---------- -----    cbbL    ----- ---------- ---------- ----------

8161 AATGTACGAG CGTGCTGAGT TCGCTAAAGA AATCGGTACT CCGATCATCA TGCACGATTA CCTAACAGGT GGTTTCACTG
     ---------- ---------- ---------- -----    cbbL    ----- ---------- ---------- ----------

8241 CTAACACAGG TCTTGCGAAC TACTGTCGTA AGAATGGTCT GTTGTTACAC ATTCACCGCG CAATGCACGG TGTAATTGAC
     ---------- ---------- ---------- -----    cbbL    ----- ---------- ---------- ----------

8321 CGTAACCCAC ATCACGGTAT TCACTTCCGT GTTTTAACGA AAGCACTACG TCTATCGGGT GGTGATCACT TACACTCAGG
     ---------- ---------- ---------- -----    cbbL    ----- ---------- ---------- ----------

8401 TACTGTTGTT GGTAAGCTTG AAGGTGACCG TGAAGCAACT CTAGGTTGGA TCGATCTTAT GCGTGATTCA TTCATTCCTG
     ---------- ---------- ---------- -----    cbbL    ----- ---------- ---------- ----------

8481 AAGATCGTTC ACGTGGAATC ATGTTCGACC AAGACTTCGG TGCGATGCCT GGTGTTATGC CAGTTGCTTC TGGTGGTATC
     ---------- ---------- ---------- -----    cbbL    ----- ---------- ---------- ----------

8561 CACGTATGGC ATATGCCAGC ACTTGTTTCT ATCTTCGGTG ATGACTCTGT TCTTCAGTTC GGTGGTGGTA CGCTAGGTCA
     ---------- ---------- ---------- -----    cbbL    ----- ---------- ---------- ----------

8641 CCCATGGGGT AACGCTGCGG GTGCGGCTGC GAACCGTGTT GCTTTGGAAG CGTGTGTACA AGCACGTAAC GAAGGTAAAG
     ---------- ---------- ---------- -----    cbbL    ----- ---------- ---------- ----------

8721 AAGTCGAGAA AGAAGGTAAA GAAATCCTTA CTAACGCTGC TAAACATAGC CCAGAACTTA AGATCGCAAT GGAAACTTGG
     ---------- ---------- ---------- -----    cbbL    ----- ---------- ---------- ----------
```

```
                                                                                    LysR bs1cbbS
8801 AAAGAAATCA AGTTCGAATT CGACACAGTT GATAAGCTAG ATGTTAAGCA TAAGTAATAG ATAATTTAGG AGAATATAAT
     ---------- ---------- -----    cbbL    ------ ---------- --------          TnA  - n8 - TnA

8881 TATGAGTATT CAAGATTACC CATCTCGTCT TTCAGATCCA CAATCTCGTA AGGCTGAGAC GTTCTCTTAC CTTCCAAAAA
      --------- ---------- ---------- -----      cbbS     ----- ---------- ---------- ----------

8961 TGACGGCTGA GCAAATCAAA GCACAAGTTC AGTACATCAT TGACAAAGGT TGGAACCCTG CAATCGAACA TTCAGAGCCA
     ---------- ---------- ---------- -----      cbbS     ----- ---------- ---------- ----------

9041 GAGAATGCGT TCTCTTACTA CTGGTATATG TGGAAGCTTC CTATGTTCGG TGAAACTGAC GCTGATGCCG TATTGGCAGA
     ---------- ---------- ---------- -----      cbbS     ----- ---------- ---------- ----------
         LysR bs1cbbQ-1
9121 AGTTGATGCA TGTATCAAAG CTAACCCAAA CAACCACGTT CGTTTGATTG GTTATGATAA CTATGCACAG TCTCAAGGTG
     ---------- ---------- ---------- -----      cbbS     ----- ---------- ---------- ----------
        TnA  - n8 - TnA                                       -35 box cbbQ-1
                                                                  ------               --
9201 CGAACATGCT TATTAAGCGT GGTGACATGT AATTTTAATT AAAATTACAT GACCTTACCC CTCAGGCGTT GCCTGAGGGG
     ----------     cbbS    - ---------- --                                      IR10
  -10 box cbbQ-1
     -------
9281 CCAAATTTTG AGGGGAGATT AAACATCGGG TTTAATGCTT CTCTCGCCCT GGTTTACTTA GGCAGCTTAT ACCTCCTGCT

                           IR11                                 LysR bs2cbbQ-1     IR12
9361 TAAGTAACGG CCAGCTAGCC CCGAAACTAG CTGGTTTTCA ATCCATTTAG ATTTACTTAT TTTAATCAAG TAATGAAATC
                                                           TnA - n7 - TnA

9441 TAAATATTTT GAATGCTACT TGCCAATGGA GAAAAAACAT GTCGGAAATT AACGCATTAG ATCCTAGTCA ATACCTTATT
                                               -- ---------- --   cbbQ-1   ------- ----------

9521 AAAGACGAGC CATTTTATCG TCCAGTTGCT GATGAAGTAG AGCTGTTTGA ATCGGGTTAT GCTTCAAGAA TGCCTATCAT
     ---------- ---------- ---------- -----      cbbQ-1    ---- ---------- ---------- ----------
                                                                                    IR13
9601 GTTAAAAGGG CCAACAGGGT GTGGTAAATC TCGTTTTGTT GAGTATATGG CACATAAACT CGGTAAGCCA TTAATCACTG
     ---------- ---------- ---------- -----      cbbQ-1    ---- ---------- ---------- ----------
```

```
                                                                                 IR13
 9681 TGGCGTGTAA TGAAGATATG ACCGCTTCAG ATTTGGTAGG TCGCTTCCTA ATCGATATTA ATGGTACGCG CTGGCAGGAT
      ---------- ---------- ---------- -----    cbbQ-1    ---- ---------- ---------- ----------

 9761 GGTCCTTTAA CCGTCGCGGC ACGTATCGGT GCCATCTGTT ATTTGGATGA GGTGGTTGAA GCCCGTCAAG ACACCACGGT
      ---------- ---------- ---------- -----    cbbQ-1    ---- ---------- ---------- ----------

 9841 TGTTATTCAC CCATTAACGG ATCACCGTCG TGTTTTACCT CTTGATAAAA AAGGTGAGTT GGTAGAAGCG CACCCTGATT
      ---------- ---------- ---------- -----    cbbQ-1    ---- ---------- ---------- ----------

 9921 TCCAGTTGGT TATTTCTTAT AACCCGGGTT ATCAGTCTAT GATGAAAGAC CTTAAGCAAT CGACGAAGCA GCGTTTCGGT
      ---------- ---------- ---------- -----    cbbQ-1    ---- ---------- ---------- ----------

10001 GGGTTTAGAT TTGACTACCC TGAAGAATCT GTTGAAGCCG ATATCGTTGC GAAAGAAGCG GGCATTGATA AAGAAACGGC
      ---------- ---------- ---------- -----    cbbQ-1    ---- ---------- ---------- ----------

10081 TGAAAAGCTG GTTCAAATTG CACAGCGTTC ACGTAACCTA AAAGGGCACG GTCTGGATGA AGGGATTTCA ACACGTCTAT
      ---------- ---------- ---------- -----    cbbQ-1    ---- ---------- ---------- ----------

10161 TGGTGTATGC GGCACAGCTA ATCAATAAAG GTGTGGATCC TAAAAAAGCG TGTACTATGG CCTTGATTAC GCCGTTGACA
      ---------- ---------- ---------- -----    cbbQ-1    ---- ---------- ---------- ----------

10241 GATGATGACG ATATTCTAGA TACTTTGTTA ACCACTGTTG ATACTTTCTT CGAGTAATAA AAATCAGCTC AGTCAACCGG
      ---------- ---------- -----    cbbQ-1    ---- ---------- -------

10321 TTTAAATTTA GGTCGCTTTT GCGGCCTAAT TTTTCACGGT GACTTGTAGA TATTATTGAT GATTTAAATA AGAGAAAGAG
                                       ------             --- ------
                                 -35 box cbbO-1       -10 box cbbO-1

10401 CTATGAGTTT TGATATTGAA GAAGTCAAAG CATCCTTGAT TGAAAGTGTG CCACAACTGG AAGAAATGCT TGATTCATTG
         --------- ---------- ---------- -----    cbbO-1    ---- ---------- ---------- ----------

10481 ATTCAAGAAG CTTCGCATTA TATGAATGAA GCCTCTCGTG AAACATGGTT GCAAAACGCA CAAGGAATTG CTTACCTAGG
      ---------- ---------- ---------- -----    cbbO-1    ---- ---------- ---------- ----------
```

```
10561 AAAAGGTCAA CAGGTTGTCG TTAGCTATCT TGAGGCAGTG CCGCAAGTTA TTGCTAGGAT TGATGATGAG ATTCTGGATG
      ---------- ---------- ---------- -----     cbbO-1     ---- ---------- ---------- ----------
                                IR14
10641 ATATCCTTGA AACCGTCATG AAATTGTCTT CGGTGACCTC TGGAGAAGTG GTGTCTCTTG TATTGGATTC TTTGCCTGTT
      ---------- ---------- ---------- -----     cbbO-1     ---- ---------- ---------- ----------
                                           IR14
10721 GTGTCTGAAA GAACAGGAGA TATCGACCTT TTAAGACAAT ATTTAGCGCT CGTTTACCAG ATTGGTTCCA AAACGCCACG
      ---------- ---------- ---------- -----     cbbO-1     ---- ---------- ---------- ----------

10801 CGGAATGCGT CCGATGTTGA GCAATATTGA CGAGTTGATG TCAAAACTGA CTGTTTCAGG CTTAAGACGC TGGGCACAGT
      ---------- ---------- ---------- -----     cbbO-1     ---- ---------- ---------- ----------

10881 GGGGTGCGCA AGCGCATGCT CGAAACTTCC AAGCTCAGAT TGATTACTTT GGACTGGCAT CGGAAGATTC CAAAGCGGTT
      ---------- ---------- ---------- -----     cbbO-1     ---- ---------- ---------- ----------

10961 TTCCAGCAAC AGCGTAAAGG CTCGTTGTTC ATCGATTATC ATCGACCAAT CAATTTTTAT TTGAGAGCTT TCTGGGCACG
      ---------- ---------- ---------- -----     cbbO-1     ---- ---------- ---------- ----------

11041 TGATTTCTTT ATTCGTCCTG CGGCGGCAGA TTACGATGAT TTTAAGCCTT ATTTTGAAAA TATGGCCATG CATCTACCTG
      ---------- ---------- ---------- -----     cbbO-1     ---- ---------- ---------- ----------

11121 ACGCATTGAA TGATTTAGGC GAGATTAAAG GGGGCGAGTT GTATCGCGCA ATGGCCGCCC ATATGGCATC GCATTTAGCG
      ---------- ---------- ---------- -----     cbbO-1     ---- ---------- ---------- ----------

11201 TATACCAAAG AAGCCATTTC GATGGAGCAG TTGAACCCCC AGCAAATGTT CTTTATTGAA TTGATTGAAG ATGCGCGTGT
      ---------- ---------- ---------- -----     cbbO-1     ---- ---------- ---------- ----------

11281 GGAATATAAC GCCATTAAAA ATTTCCCAGG CCTGAAGGGG CTATGGAAAA AAGTCATTAA AGCCAGTATG GAAGCCTCCG
      ---------- ---------- ---------- -----     cbbO-1     ---- ---------- ---------- ----------

11361 AGCTGCCTGA AAAGTCGACC GCTTATCGCT TAGAACAGCT TGCACTGAAA TTGATGGATG TTAAGCATGA TTTGCAAGAT
      ---------- ---------- ---------- -----     cbbO-1     ---- ---------- ---------- ----------
```

```
11441 GAGCAAATGA TGGTGGTGGC GGAACGTTTC CATAATGAAA TTGAAGAAAA TCTGGACAAC GAGAAATGGT CTTGGGATTT
      ---------- ---------- ---------- -----     cbbO-1     ---- ---------- ---------- ----------

11521 GGGGATTCTG CTTTACAATG TCTTGAATAA AGCGACTTCT AAGTGGGAAT CTCTTACGGA GATCAGTCAA CAACGTTTTG
      ---------- ---------- ---------- -----     cbbO-1     ---- ---------- ---------- ----------

11601 GCTATCGTGA TGACAACCGT TTGGTTTGGG CATCCGACGA ATGGGCCGAA ATGGAAGGGG GCGGTGCACC ACATCAAGAA
      ---------- ---------- ---------- -----     cbbO-1     ---- ---------- ---------- ----------
                                 IR15
11681 ACCGTTCGTA AAAATGTTTC ATTGATGGAA ATGATCAATG AAATTGATTC TGAACTGGTG GATGTGGATC ATGAAGAAGT
      ---------- ---------- ---------- -----     cbbO-1     ---- ---------- ---------- ----------
                                                                            IR16
11761 TTGGGTACTA GGCTCGGAGC TTTATCCTTA CGAGGATAAT GGACTGTCTT ATAATGAGAT GGAAGGCATT GAGCCCGTTT
      ---------- ---------- ---------- -----     cbbO-1     ---- ---------- ---------- ----------

11841 CAGATCCTTT CCATTATCAT GAGTGGGATT ACCGCGTTCA GTTGAATCGT CCAAACTGGG TCACGCTGTA TGAGCACCGT
      ---------- ---------- ---------- -----     cbbO-1     ---- ---------- ---------- ----------

11921 GCGAAAAAAG GCGACCCTCA GTTATATAAC CGAATTCTAG ATCAGAATAA AGGAATTGCG CATCGTATTA AGCAAATCGT
      ---------- ---------- ---------- -----     cbbO-1     ---- ---------- ---------- ----------
                                                                           IR17
12001 TGATAAGTTG CAAGCGGTTG GTTTGCAGCG TATTCGCCGA ATTGAAGATG GAGACGAACT GGATTTAAAT GCCTGCGTTG
      ---------- ---------- ---------- -----     cbbO-1     ---- ---------- ---------- ----------

12081 AAGCAATTAC GTCAATTCGT ATGGGGCATG AACCTGATCC TCGCATTACG ATGAAAAATG TGATTCGCAG CCGTGAAGTA
      ---------- ---------- ---------- -----     cbbO-1     ---- ---------- ---------- ----------

12161 TCGGTGGTCG TGTTGCTTGA TTTATCAGAA TCGACTAATG AAATGGTTGA TGGTGGCGAT AAAACTGTCC TAGAAGTAAC
      ---------- ---------- ---------- -----     cbbO-1     ---- ---------- ---------- ----------

12241 GCAGGAGGCG GCAATCTTGG TGTCGCATGC CATCAATGGT ATCGGCGATA AGTTTGCTGT TCATGGTTTC TCATCAGATG
      ---------- ---------- ---------- -----     cbbO-1     ---- ---------- ---------- ----------
```

```
12321 GGCGTCATGA CTTGCAATAC ACTCGATTCA AACAGTTTGA CGAACCTTTT GATCAAGATG TTCATTCACG TCTTGCGGGA
      ---------- ---------- ---------- -----    cbbO-1    ---- ---------- ---------- ----------

12401 ATGAAAGGTG GCCTCTCAAC TCGTATGGGC GGCGCCATGC GCCATGCGGG TAGTTACCTT GAGAAACAAT CCAGTAAGCA
      ---------- ---------- ---------- -----    cbbO-1    ---- ---------- ---------- ----------
                                                             IR18
12481 GAAGCTGTTG TTGGTCATTA CCGATGGTGA ACCAGCGGAT ATTGACGAAA AAGACGGTCA ATATTTAAAA CAAGATGCGA
      ---------- ---------- ---------- -----    cbbO-1    ---- ---------- ---------- ----------

12561 AGAAAGCCGT TGAAGAACTT CAGGCGAAAG GGGTTTATTC GTATTGCTTA ACGATCGATC AGTATGCCGA TAAGTATGTG
      ---------- ---------- ---------- -----    cbbO-1    ---- ---------- ---------- ----------

12641 CATAATATTT TTGGTCAAAA CCGTTATGCG ATTGTGGATA ATGTCTTGAA GCTGCCTGAA AAATTACCGC AATTATTTGC
      ---------- ---------- ---------- -----    cbbO-1    ---- ---------- ---------- ----------

12721 AAACTTAACC ACCTAACTTG GAACGAAAAA TAAGATGAAA GCACAACCGT TTATTGAAGC CGTCAAGCAG TTAACTGATG
      --   cbbO-1   ----                     ------ -------  hypothetical protein   ----------

12801 ATGATTTTCA GCTTATTCTT GAAGGAAGTG CCATTATCAT TGAAAATGAT GTGGCATTAA CAACAGGAAG GGCCGACAGT
      ---------- ---------- --------   hypothetical protein   --------- ---------- ----------

12881 GCTTATGTTA TTTATGAACT GGGAGAAGAC CCTTTTACAT CTTCTGACGA GATTAAAGCG TTTTTAATAC AGAATGCCGA
      ---------- ---------- --------   hypothetical protein   --------- ---------- ----------

12961 AGCGCTTTTA AAAGAGTATT ACCAGTTTAA TCCGGTCAGC CGTCAATATT TTGATCGCAG TTT
      ---------- -----   hypothetical protein   - ---------- ---------- ---
```

Structural features predicted for the DNA of the metagenome derived RubisCO gene cluster. Exact positions of predicted promoter boxes are indicated by black dashes over each associated base and a heading with -10 box or -35 box. Putative LysR binding sites (bs) are highlighted in green. Inverted repeats (IR) are numbered consecutively from IR1 to IR18. Each IR is highlighted in grey and red, whereby the grey part shows the repeat and its reverse complement, putatively forming the stem, and the bases in between, in red, represents the putative loop forming area. Only IR with at least 8 bp in the potential stem region and loops with a maximum of 100 nt were considered.

Supplementary Figure 3

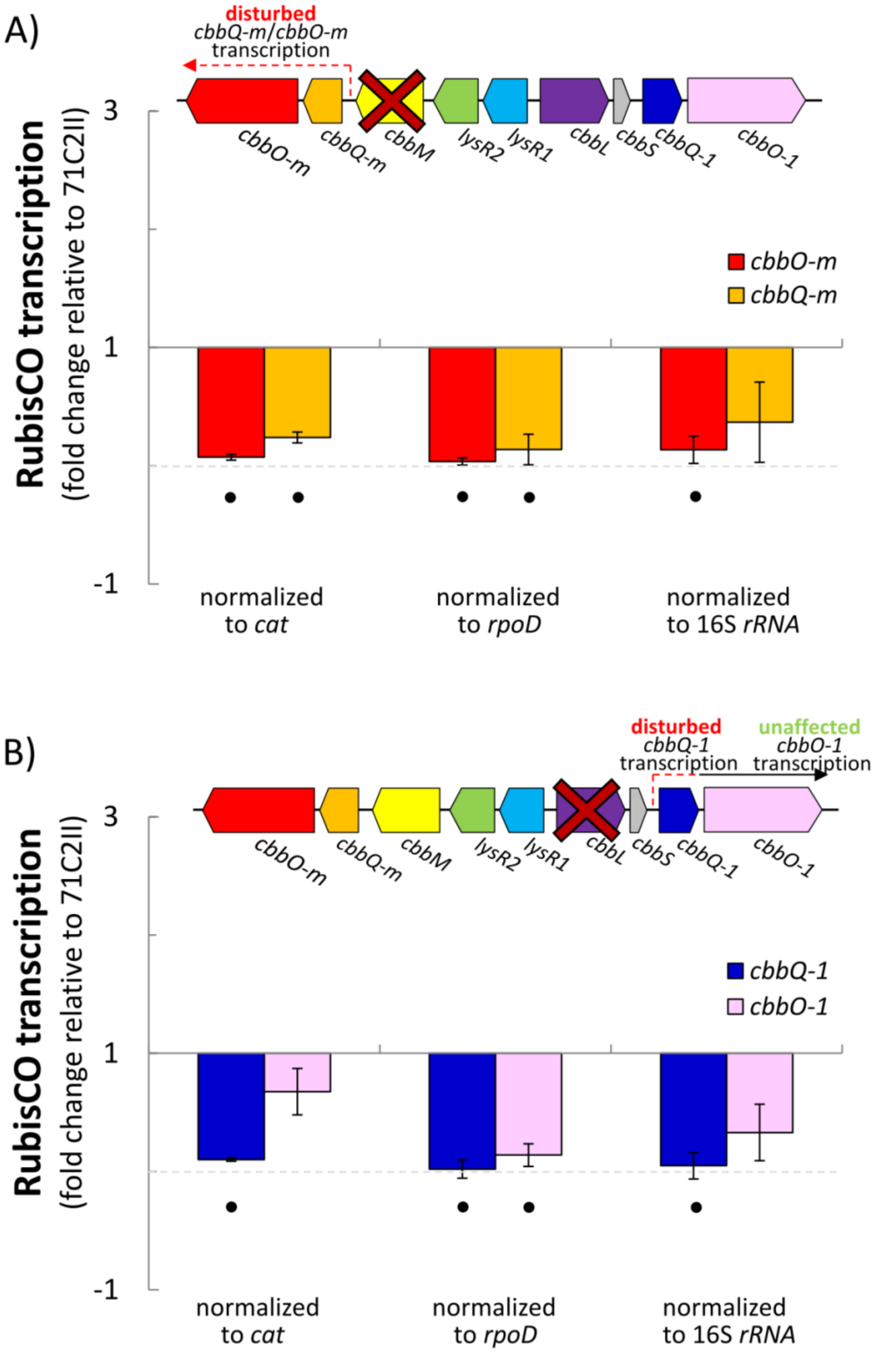

Fold changes of *cbbO-m, cbbQ-m*, *cbbQ-1* and *cbbO-1* transcription. Fold changes ($2^{-\Delta\Delta Ct}$) of A) *cbbO-m* (red) and *cbbQ-m* (orange) transcripts expressed from the transposon clone Δ*cbbM* (22II) and of B) *cbbQ-1* (blue) and *cbbO-1* (pink) transcripts expressed from the transposon clone Δ*cbbL* (24II). The data shown is normalized to three different reference genes, the *cat* gene, which is encoded on the fosmid vector and reflects its copy number, the 16S rRNA and *rpoD* housekeeping genes. RubisCO transcription is relative to the clone 71C2II, containing the intact RubisCO gene cluster (*cbbO-mQ-mM lysR2lysR1 cbbLSQ-1O-1*). Bars and error bars denote mean values and +/- standard errors. Black dots denote that values are significantly different (p-value ≤0.05).

Supplementary Tables

Supplementary Table 1

Supplementary Table 1. Insertion positions of tested double transposon clones with 13 kb inserts.

**inserted genes**	**clone designation**	**insertion position [aa]**	**total orf length [aa]**	**specific RubisCO activity [nmol 3-PGA*min^{-1}*mg^{-1}]**
/	71C2II	/	/	49 ± 6
Δ*cbbM*	22II	167	459	265 ± 48
Δ*cbbM* Δ*cbbO-m*	22II1A3	208	757	130 ± 22
	22II1G1	34		178 ± 24
Δ*cbbM* Δ*cbbQ-m*	22II2H4	135	266	81 ± 7
Δ*cbbM* Δ*lysR2*	22II3A3	28	314	126 ± 39
Δ*cbbM* Δ*lysR1*	22II2B2	79	308	163 ± 29
Δ*cbbM* Δ*cbbL*	22II1C5	15	472	0.5 ± 7
	22II2B4	377		-1.5 ± 11
Δ*cbbM* Δ*cbbQ-1*	22II2C9	159	272	205 ± 48
Δ*cbbM* Δ*cbbO-1*	22II2A10	75	777	235 ± 32
	22II4H6	503		234 ± 10
Δ*cbbL*	24II	38	472	18 ± 7
Δ*cbbL* Δ*cbbO-m*	24II2H3	482	757	17 ± 5
	24II2G7	212		13 ± 5
	24II1H11	82		14 ± 5
Δ*cbbL* Δ*cbbQ-m*	24II1F9	147	266	17 ± 5
Δ*cbbL* Δ*cbbM*	24II2F3	301	459	-4 ± 5
Δ*cbbL* + Δncr*cbbM-lysR2*	24II1G2	103[1]	214[2]	8 ± 3
Δ*cbbL* Δ*lysR2*	24II1H7	46	314	48 ± 8
Δ*cbbL* Δ*lysR1*	24II2F11	263	308	16 ± 7
	24II1H1	234		17 ± 7
Δ*cbbL* + Δncr*cbbS-cbbQ-1*	24II2G1	222[1]	245[2]	9 ± 2
Δ*cbbL* Δ*cbbQ-1*	24II6H6	10	272	2 ± 2
	24II5G11	43		6 ± 1
Δ*cbbL* Δ*cbbO-1*	24II6G12	367	777	17 ± 3
	24II5G9	416		19 ± 4
	24II5D3	618		13 ± 1

[1] insertion positions in non-coding regions given in nucleotides
[2] total length of non-coding region given in nucleotides
